# Mutational Signature and Integrative Genomic Analysis of Human Papillomavirus-Associated Penile Squamous Cell Carcinomas from Latin American Patients

**DOI:** 10.3390/cancers14143514

**Published:** 2022-07-20

**Authors:** Luisa Matos do Canto, Jenilson Mota da Silva, Patrícia Valèria Castelo-Branco, Ingrid Monteiro da Silva, Leudivan Nogueira, Carlos Eduardo Fonseca-Alves, André Khayat, Alexander Birbrair, Silma Regina Pereira

**Affiliations:** 1 Clinical Genetics Department, University Hospital of Southern Denmark, 7100 Vejle, Denmark; luisaaamatos@gmail.com; 2 Postgraduate Program in Health Science, Federal University of Maranhão, São Luís 65080-805, MA, Brazil; jenilson.motadasilva@gmail.com; 3 Laboratory of Genetics and Molecular Biology, Department of Biology, Federal University of Maranhão, São Luís 65080-805, MA, Brazil; patricia.valeria@ufma.br (P.V.C.-B.); ingrid.monteiro@discente.ufma.br (I.M.d.S.); 4 Aldenora Bello Cancer Hospital, São Luís 65031-630, MA, Brazil; leudivan.nogueira@gmail.com; 5 Health Science Institute, Paulista University, Bauru 186085-500, SP, Brazil; carloseduardofa@hotmail.com; 6 Oncology Research Center, Federal University of Pará, Belém 66073-005, PA, Brazil; andresk@ufpa.br; 7 Department of Dermatology, School of Medicine and Public Health, University of Wisconsin-Madison, Madison, WI 53706, USA; birbrair@wisc.edu; 8 Department of Pathology, Federal University of Minas Gerais, Belo Horizonte 31270-901, MG, Brazil; 9 Department of Radiology, Columbia University Medical Center, New York, NY 10032, USA

**Keywords:** whole-exome sequencing, HPV, penis, carcinoma, biomarkers

## Abstract

**Simple Summary:**

DNA sequencing has been crucial to comprehending cancer mutational patterns, leading to the identification of driver genes and altered signaling pathways. Thus, identifying new pathogenic variants and their impact on tumor onset, progression, and treatment response has fueled tumor biology research. Here, we present novel findings addressing the first whole-exome sequencing (WES) of human papillomavirus (HPV)-associated penile squamous cell carcinoma (PSCC) from Latin Americans and its association with pathogenesis. We also compared the molecular profile of the tumors to that of three previous studies from populations with different genetic and socioeconomic backgrounds, the majority of which was HPV-negative. We describe the most altered genes and the main pathogenic variants found in the Latin Americans, ten of which are exclusive to our study sample. The data allowed us to identify molecular pathways and druggable targets with potential treatment value for this still-neglected HPV-associated carcinoma.

**Abstract:**

High-throughput DNA sequencing has allowed for the identification of genomic alterations and their impact on tumor development, progression, and therapeutic responses. In PSCC, for which the incidence has progressively increased worldwide, there are still limited data on the molecular mechanisms involved in the disease pathogenesis. In this study, we characterized the mutational signature of 30 human papillomavirus (HPV)-associated PSCC cases from Latin Americans, using whole-exome sequencing. Copy number variations (CNVs) were also identified and compared to previous array-generated data. Enrichment analyses were performed to reveal disrupted pathways and to identify alterations mapped to HPV integration sites (HPVis) and miRNA–mRNA hybridization regions. Among the most frequently mutated genes were *NOTCH1*, *TERT*, *TTN*, *FAT1*, *TP53*, *CDKN2A*, *RYR2*, *CASP8*, *FBXW7*, *HMCN2*, and *ITGA8*. Of note, 92% of these altered genes were localized at HPVis. We also found mutations in ten novel genes (*KMT2C*, *SMARCA4*, *PTPRB*, *AJUBA*, *CR1*, *KMT2D*, *NBEA*, *FAM135B*, *GTF2I*, and *CIC*), thus increasing our understanding of the potential HPV-disrupted pathways. Therefore, our study reveals innovative targets with potential therapeutic benefits for HPV-associated PSCCs. The CNV analysis by sequencing (CNV-seq) revealed five cancer-associated genes as the most frequent with gains (*NOTCH1*, *MYC*, *NUMA1*, *PLAG1*, and *RAD21*), while 30% of the tumors showed *SMARCA4* with loss. Additionally, four cancer-associated genes (*CARD11*, *CSMD3*, *KDR*, and *TLX3*) carried untranslated regions (UTRs) variants, which may impact gene regulation by affecting the miRNAs hybridization regions. Altogether, these data contribute to the characterization of the mutational spectrum and its impact on cellular signaling pathways in PSCC, thus reinforcing the pivotal role of HPV infection in the molecular pathogenesis of these tumors.

## 1. Introduction

Several genes are frequently mutated in various cancers, yet the altered gene spectra vary greatly [1]. The advancement of high-throughput DNA sequencing has been crucial to comprehending cancer mutational patterns, leading to the identification of driver genes and altered signaling pathways. This knowledge enables the association of mutational profiles with specific tumors and their clinical–histopathological parameters. Thus, identifying new pathogenic variants and their impact on tumor onset, progression, and treatment response has fueled tumor biology research [2]. Here, our goal was to characterize the mutational profile of HPV-positive PSCC and its association with pathogenesis.

Penile cancer (PeCa) is one of the most neglected malignancies; it is described as a rare cancer in high-income nations, but it has higher incidences in developing countries. This can be partly attributed to social and economic issues, such as poor genital hygiene, lack of childhood circumcision, phimosis, and chronic inflammation. In addition, human papillomavirus (HPV) infection, often linked to unprotected sex with multiple partners, has been identified as a risk factor for penile carcinogenesis [3]. In some regions of Brazil, PeCa may account for up to 10% of male cancers [4,5,6]. Notably, the PeCa incidence is progressively increasing even in developed countries, along with HPV infections and oropharynx cancer [7,8,9].

Our previous studies corroborate the role of HPV in PeCa, showing a high frequency of cytobands with copy number alterations (CNAs) harboring HPVis, as well as the impact of miRNA/mRNA changes on HPV-related molecular pathways [10,11,12]. However, the molecular characterization of these tumors, especially upon HPV infection, is still limited. Furthermore, patients’ treatments disregard HPV status, though infection contributes to PeCa classification [13]. Surgical resection and systemic chemotherapy are the primary treatment approaches. Nevertheless, they result in poor outcomes, low survival, and a high risk of comorbidities and psychosocial issues [3]. Information about the somatic mutation landscape of PeCa is limited to patients from Europe, Asia, and North America [14,15,16], who are mostly HPV-negative. Further investigation is needed to unravel HPV-associated molecular alterations and identify potential targets for more efficient personalized treatments.

In the present work, we report unique data on the mutational signature of HPV-associated PSCC from Latin American patients. We described novel genes and pathogenic variants that may contribute to a better understanding of the potential HPV-disrupted pathways. This study reveals innovative targets with potential therapeutic benefit for this carcinoma.

## 2. Materials and Methods

Supplementary Figure S1 shows the flowchart representing the study design.

### 2.1. Study Population

Thirty patients with PSCC underwent chemotherapy-naive surgical excision at the Aldenora Bello Cancer Hospital (São Luís, Maranhão, Brazil). All patients submitted written informed consent, which was approved by the Research Ethics Committee on Humans from the Federal University of Maranhão and by the National Research Ethics Commission (CONEP, Brazil; CAAE: 46371515.5.0000.5087). Clinical and histopathological variables were extracted from the patients’ medical records.

At diagnosis, the mean age of the patients was 60.4 ± 16.4 years old, ranging from 31 to 85 years old. Most patients presented a low level of education (83.3% had no studies or incomplete primary education, and only one had an undergraduate degree), 43.3% self-declared themselves to be a current smoker or declared to have been a smoker in the past 5 years, and 36.7% declared that they drink alcohol regularly. Additionally, 62.5% of the patients presented phimosis. All tumors were SCC, presenting an average size of 4.8 ± 2.7 cm. The tumors were localized in the glans, corpus cavernosum, and corpus spongios in 20% of the cases. The most frequent histological subtypes were condylomatous and usual, which were present in 46.7% and 40% of the cases, respectively. Tumor grades G2 and G3 were the most frequent, present in 66.7% and 26.7% of the patients, respectively. Partial and total penectomy were performed in 63% and 30% of the patients, respectively, while lymphatic and perineural invasion were performed in 18.5% and 33.3%, respectively. Table 1 and Supplementary Table S1 present the detailed patients’ clinical–histopathological information.

### 2.2. HPV Genotyping

We performed HPV genotyping via nested-PCR according to BioGenetics Molecular Technologies (Uberlândia, Minas Gerais, Brazil, patent number BR102017004615.0), which allowed us to identify 40 viral subtypes classified as high risk (16, 18, 31, 33, 35, 39, 45, 51, 52, 56, 58, 59, 66, 68, 73, and 82), intermediate risk (MM7, MM8, 26, 30, 34, 53, 54, 55, 61, 62, 64, 67, 69, 70, 71, 72, 74, and 81), and low risk (6, 11, 42, 43, 44, and 57). In addition, we performed DNA sequencing according to our previous protocol [10].

### 2.3. Whole-Exome Sequencing

Genomic DNA was isolated from 30 surgically fresh tumor tissues that were macro-dissected by an experienced pathologist and presented at least 80% of tumor cells. Twenty of them were paired with nontumor tissue located roughly two centimeters away from the primary tumor.

DNA from tumor and nontumor tissues was extracted by using DNeasy^®^ Tissue Kit (QIAGEN Group, Germantown, MD, USA), according to the manufacturer’s protocol. DNA library was prepared by using 100 ng input gDNA of each tumor and their matched adjacent nontumor samples. DNA was fragmented by using a Covaris ME220 ultrasonicator and quantified on Qubit (Invitrogen, Waltham, MA, USA) according to the manufacturer’s instructions. Library preparation was performed by using the Illumina^®^ TruSeq^®^ Exome Kit (Illumina, San Diego, CA, USA) and IDT custom probe enrichment (Integrated DNA Technologies, Coralville, IA, USA), according to the manufacturer’s guidelines. The size, quantification, and quality control of the DNA library were evaluated on TapeStation (Agilent Technologies, Santa Clara, CA, USA). The captured DNA was subjected to high-performance sequencing, using Illumina NovaSeq 6000 platform to generate 2 × 150-bp paired-end reads.

### 2.4. Data Processing

To explore the spectrum of somatic variants, three pipelines were developed by VarStation Bioinformatics (https://varsomics.com/varstation/ (last accessed on 10 May 2022)), following the steps described below. The quality control of the raw sequence reads and the presence of adapters were evaluated by FASTQC v0.11.9 (https://qubeshub.org/resources/fastqc (accessed on 22 March 2021)) and MultiQC v.1.10.1 [17]. Regions with low quality and containing adapters were trimmed, and the resulting sequences that were smaller than 50 base pairs were excluded by using Cutadapt v2.10 [18]. The FASTQ files were aligned to the reference human genome (hg38), using BWA-MEM v.0.7.13 [19]. The aligned BAM files were ordinated according to genomic annotation, PCR duplicates were removed, and the outputs were realigned by using the Indel Realignment tool of the Genome Analysis Toolkit (GATK) [20]. The recalibration was carried out by using the BaseRecalibrator tool from GATK. Moreover, the GATK Crosscheck Fingerprints tool was used to identify possible exchange between the tumor/nontumor sample pairs. The high-performance sequencing produced 73.78 ± 17.4 million and 75.35 ± 18.2 million of paired-reads for the tumors and nontumor samples, respectively, both with 99.68% ± 0.1% of DNA mapping on target. The mean coverage was 294× and 300× for tumor and nontumor tissues, with 93% ± 6% and 94% ± 8% of target DNA covered at least 100×, respectively (Supplementary Figure S2). All sequenced samples presented > 95% of quality score ≥ Q30 (mean equals to 97% ± 0.5% for tumor and 96.7% ± 0.7% for nontumor) (Supplementary Table S2).

### 2.5. Variant Calling

The somatic and germline variant calling was performed by using the best practice of the GATK4 [21]. The HaplotypeCaller [22] was used for germline variants, and the Mutect2 tool [23] was used for somatic variants.

The normal panel was built with the 20 adjacent nontumor samples, which were paired to their respective tumor samples (somatic paired samples) to identify single-nucleotide variants (SNVs) and nucleotide (s) deletion coupled with a nucleotide (s) insertion (Indels). In addition, the 10 tumor samples without corresponding nontumor tissue (Somatic single samples) followed the tumor-only pipeline. For comparison purposes, the somatic paired samples were also analyzed as tumor-only, totaling 30 samples. The comparison of VCFs files obtained from these different analyses was performed by using vcfeval [24], which revealed 82% matching between somatic paired and somatic single samples, while there was no matching between somatic paired and germline samples, thus confirming the usefulness of the pipeline for somatic variants through 20 paired samples.

### 2.6. Variant Filtering

Each sample was evaluated individually by using the VarStation platform (https://app.varstation.com/#/analysis (last accessed on 10 May 2022)), following predetermined parameters: read depth ≥ 100, variant allele fraction (VAF) ≥ 0.05, and populational frequency < 0.01 (gnomAD, 1000 Genomes and ABRAoM, the Brazilian genomic variants database (https://abraom.ib.usp.br (accessed on 22 March 2021))). We also performed a visual analysis by using the Integrative Genomics Viewer (IGV, Broad Institute and UC San Diego) to filter out variants present in problematic regions of the genome [25]. The variant calling covered SNVs and Indels at exonic, intronic, or intergenic regions, such as splicing, frameshift, non-frameshift, non-coding RNA (ncRNA), UTR, stop-gain, stop-loss, start-gain, and start-loss.

Both synonymous and nonsynonymous variants that were predicted to be benign or probably benign were filtered out by the predictor’s algorithms, including SIFT, LRT, Revel, MutationTaster, MutationAssessor, Fathmm-XF, Fathmm-MKL Vest, MetaSVM, Polyphen2, Polyphen2 HDIV, HVAR, CADD, DANN, MetaLR, MetaSVM, and Provean. Clinical information was extracted from ClinVar [26], and data on cancer-related mutations were obtained from the Catalogue of Somatic Mutations in Cancer (COSMIC) at https://cancer.sanger.ac.uk/cosmic (accessed on 22 March 2021) and OncoKB (https://www.oncokb.org (accessed on 22 March 2021)). The variants obtained were annotated using by ANNOVAR [27].

Following the analysis of the 20 paired tumors, only the altered genes occurring in at least 15% of the cases were further analyzed in the 10 single tumors. In addition, we looked at the variants, especially the ones classified as pathogenic or likely pathogenic according to ClinVar (http://www.ncbi.nlm.nih.gov/clinvar (accessed on 22 March 2021)), following the somatic variant interpretation/reporting by the American College of Medical Genetics and Genomics (ACMG), Association for Molecular Pathology (AMP), and College of American Pathologists (CAP) recommendations [28,29]. All variants were searched in the COSMIC v9, released 28 May 2021 (https://cancer.sanger.ac.uk/cosmic (accessed on 1 June 2021)), and in dbSNP (https://www.ncbi.nlm.nih.gov/snp/ (accessed on 1 June 2021)). Our data were compared to variants described for penile tumors from European [14], Asian [15], and North American [16] populations containing mostly HPV-negative.

### 2.7. Copy Number Variation by Sequencing (CNV-seq)

The 20 paired samples and the 30 single tumor samples (20 paired plus 10 unpaired) were also used for calling up somatic CNVs, using high-throughput sequencing. We followed the Best Practices guide GATK4, using the somatic pipeline for CNV-seq [21]. The normal panel was built with the same samples as for SNV/Indels. Variants were annotated with Funcotator v1.6.20190124 s and AnnotSV v2.5 [30]. We considered cutoffs for calling gain and deletion as CN > 3 and CN < 1, respectively. In addition, the specified regions should have over 10 Kb and should be present in at least 10% of samples [31].

### 2.8. UTR-Perturbing Variants

We used the miRabel platform [32] to identify miRNAs that regulate genes with variants in UTRs. Prediction tools available in miRabel, such as MiRanda, Pita, SVmicro, and TargetScan, were used. Only miRNA–mRNA interactions with a score ≤ 0.05 were considered. The miRNA–mRNA interactions identified in miRabel were then used in the STarMir program [33] to identify the seed regions. The construction of the design and nucleic acid fold (STarMir) is obtained by using the Mfold package [34] and Sfold containing the Srna module [35]. Sfold applies a two-step model for hybridization between mRNA and miRNA. In this model, the hybridization of the miRNA–target occurs at an accessible target site, and then the hybrid elongates to form the complete miRNA–target duplex. The minimum free energy of hybridization was obtained from the RNAhybrid tool [36,37]. Only interactions with LogitProb values ≥ 0.5 were considered.

### 2.9. Integrated Genomic and Molecular Characterization of HPV-Associated PSCC

The thirty sequenced tumor samples were analyzed by qPCR for alterations in gene copy number and/or gene expression, while 18 tumors were evaluated for CNA by aCGH [10]. The data were cross-referenced to produce an overview of the main genes with SNV/Indels, CNV-seq, CNA, and gene expression changes (GECs) in HPV-associated PSCC in the Latin Americans.

### 2.10. Pathway Analysis

The Integrative Onco Genomics (IntOGen) platform [38] was used to identify variants of cancer-related genes in both paired and unpaired tumors, which were confirmed by using COSMIC [39]. The STRING v.11.5 database [40] was used to predict protein–protein interactions (PPI) based on direct (physical) and indirect (functional) associations (score > 0.9). The KEGG pathways (Kyoto Encyclopedia of Genes and Genomes) were used for functional analysis through the enrichment of STRING clusters (FDR < 0.05, with *p*-values corrected by the Bonferroni method), which generate sets of functionally associated proteins at various levels of hierarchy, ranging from small groups of 5 proteins to large sets of up to 200 proteins [41].

## 3. Results

### 3.1. High Frequency of HPV Infection in PSCC

All patients (n = 30) were tested for HPV infection by nested-PCR and direct DNA sequencing, revealing 100% of positivity for the virus (Table 1). Genotyping was possible in all but five samples, which yielded insufficient DNA. High-risk HPV subtypes were detected in 96% of the tumors, of which 29.2% presented multiple infections. The HPV16 was the most frequent (75%).

### 3.2. Somatic Variants in HPV-Associated PSCC in Latin Americans

**Figure 1:**
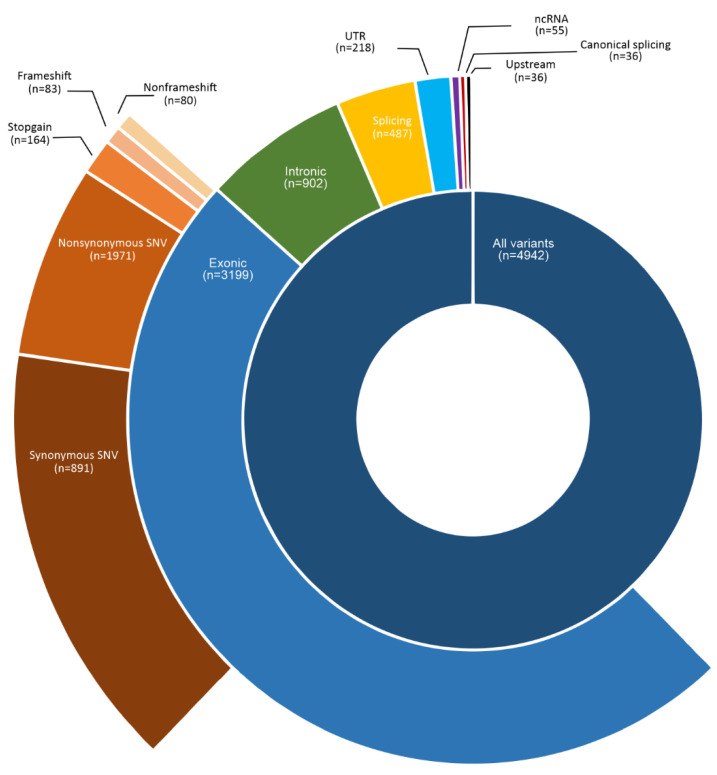
Somatic variants identified in human papillomavirus-associated penile squamous cell carcinomas from Latin American patients. The full circle in darker blue represents the total number of variants identified in 3872 genes. The semicircle in the middle represents the number of variants identified according to the gene’s location: exonic (2654 genes), intronic (850 genes), splicing (466 genes), UTR (214 genes), ncRNA (48 genes), canonical splicing (35 genes), and upstream (28 genes). The outer semicircle shows the types of variants identified in the exonic region: synonymous SNV (853 genes), non-synonymous SNV (1742 genes), stop-gain (149 genes), frameshift (73 genes), and non-frameshift (77 genes).

The WES of 30 PSCC and 20 matched adjacent normal penile tissues produced 149.14 million reads with high-quality control (Supplementary Figure S2). Firstly, the variant calling was performed in the 20 paired tumors. Considering the parameters described in the variant calling (see Materials and Methods), we identified 4942 variants affecting 3277 genes, most of them (64.73%) at exonic regions, of which 96% were SNV and 4% Indels (Figure 1). The most frequent alteration was nonsynonymous SNV (61.6%) due to transitions C > T (28.3%) and G > A (27.2%), while start loss (n = 8) and stop-loss (n = 2) ones were the less frequent. We also identified variants in intronic region (18.25%), splicing sites (9.85%), UTR (4.4%), and ncRNA (1%). Less frequent variants were in upstream (n = 36), intergenic (n = 7), and downstream (n = 2) regions. As expected, the largest human chromosome (chr1) presented most of the variants (11%), followed by chromosomes 19 and 2 (7% each). Chromosome Y had only two variants.

**Figure 2:**
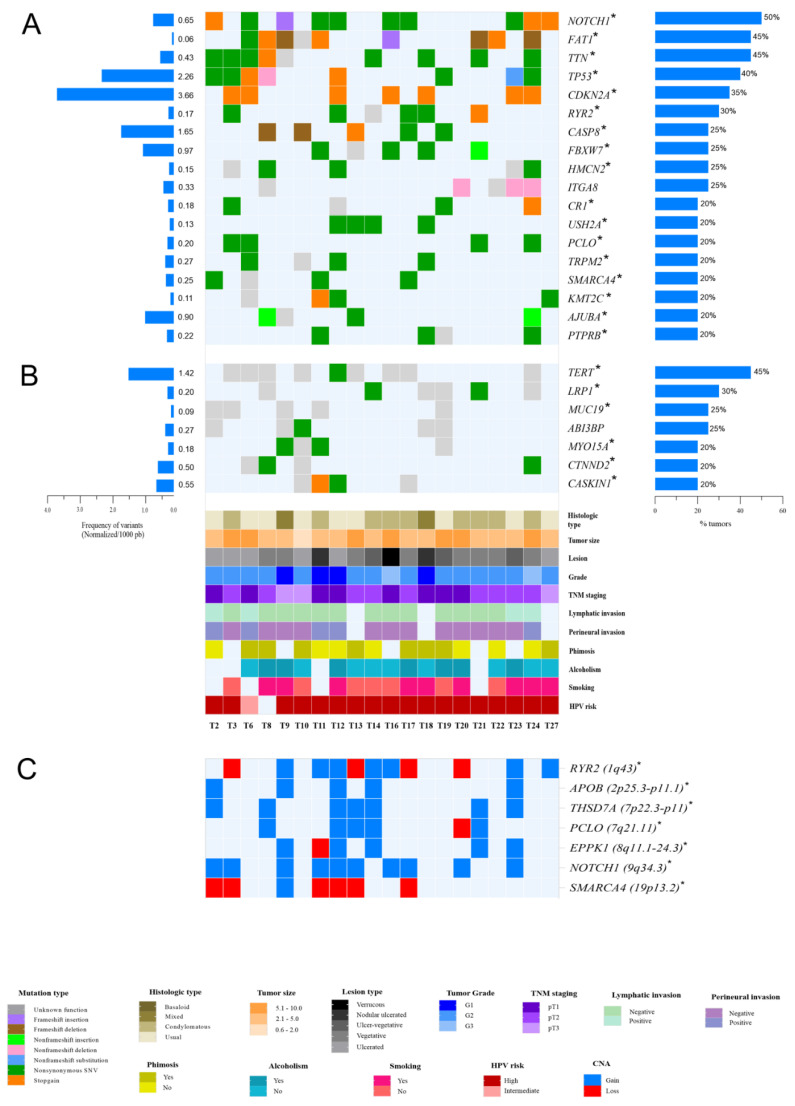
Top genes and variants identified in at least 20% of human papillomavirus-associated penile squamous cell carcinomas according to clinical and histopathological variables. The charts in (**A**) (exonic region), (**B**) (intronic region), and (**C**) (CNV-seq) present the mutational profile of the 20 paired tumors. The matrix at the bottom of (B) shows the clinical–histopathological profile and the HPV status of each case (identified by the samples ID). Horizontal bar plot on the right shows the frequency of each altered gene in the 20 paired tumors, and on the left is the number of variants normalized according to the gene size (1000 bp). * Genes with variants localized at HPV integration sites; CNV-seq, copy number variation by sequencing; CNA, copy number alterations.

Among 3277 genes presenting rare variants, 160 have been reported as cancer-associated genes according to Integrative Onco Genomic (https://www.intogen.org/ (accessed on 14 October 2021) [38], as shown in Supplementary Table S3). The number of cancer-associated genes per tumor was 12 ± 7. Supplementary Table S4 shows the top mutated genes (n = 84) found in at least 15% of the tumors, and their normalized score according to the gene size (bp), of which 18 (21.43%) are cancer-associated genes (*NOTCH*, *FAT1*, *TP53*, *CDKN2A*, *FBXW7*, *CASP8*, *SMARCA4*, *PTPRB*, *KMT2C*, *CR1*, *AJUBA*, *KMT2D*, *MTOR*, *NBEA*, *LRP1B*, *FAM135B*, *GTF2I*, and *CIC*). Figure 2A presents the most frequent (>20% of the tumors) genes with exonic variants (n = 18). *NOTCH1* was the most frequent (50% of the tumors), followed by *TTN* and *FAT1* (45% each); *TP53* (40%); *CDKN2A* (35%); *RYR2* (30%); *CASP8*, *FBXW7*, *HMCN2*, and *ITGA8* (25% each); *CR1*, *USH2A*, *PCLO*, *TRPM2*, *SAMARCA4*, *KMT2C*, *AJUBA*, and *PTPRB* (20% each). Considering the number of variants according to the gene size, *CDKN2A*, *TP53*, *CASP8*, *FBXW7*, and *AJUBA* are the hotspots. Among the most frequent genes presenting non-exonic alterations (n = 7), we observed notable somatic variants (45% of the tumors) in the *TERT* gene (Figure 2B). We highlight that 92% (23/25) of the most frequent mutant genes carry variants at HPVis.

For the most frequently altered genes, the variants occur in a single tumor, such as *NOTCH1* and *TP53*. We highlight genes having repeated variants, such as *CDKN2A*. This gene presented only pathogenic variants, all of them stop-gain, of which c.238C > T (p.Arg80Ter) and c.172C > T (p.Arg58Ter) were observed in 15% and 10% of tumors, respectively, in addition to c.330G > A and c.329G > A (p.Trp110Ter). The *FBXW7* gene presented the variant c.1273A > G (p.Arg425Gly) in 10% of the tumors. Furthermore, some variants were found in both groups of tumors, paired and single, such as c.330G > A (*CDKN2A*), c.4389C > A (*NOTCH1*), and c.743G > A (*TP53*), each present in 6.7% of the tumors. Among the 229 variants detected in the main altered genes, including those found exclusively in Latin Americans, 123 (57.7%) are not described in COSMIC v9 or in the dbSNP (Supplementary Table S5).

We also highlight the occurrence of 10 (50%) variants in *TERT* (telomerase catalytic subunit reverse transcriptase), of which one occurs in exonic region (c.2713G > A; p.Val905Met) and nine (45% of tumors) in the upstream region of the gene. All *TERT* promoter (*TERT*p) variants were G > A transitions, with 66.7% of them being located at −146bp (rs1561215364). This variant was also found in 50% of the single tumors. The second most frequent (10% of paired tumors) was at −124bp (rs1242535815).

### 3.3. Novel Genes Altered Exclusively in PSCC from Latin Americans

**Figure 3:**
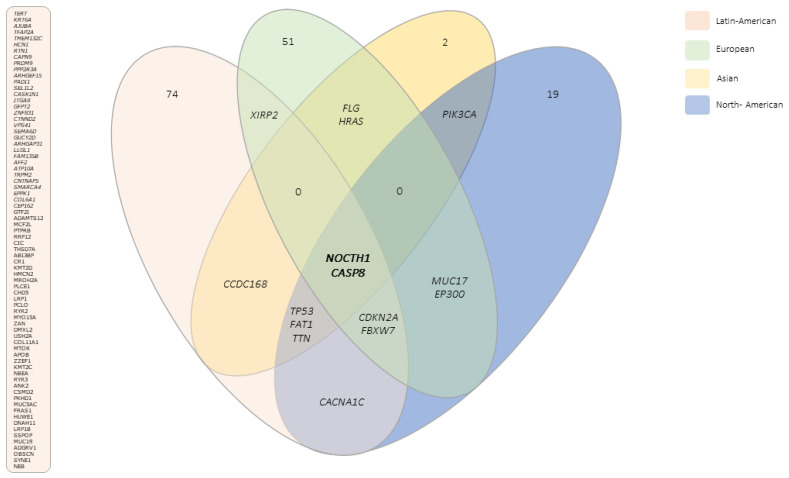
Common genes described in four studies in which whole-exome sequencing was performed in penile carcinomas, including the present study. Data from Europeans, Asians, and North Americans were adapted from [14,15,16], respectively. Left side of the figure: genes observed exclusively in penile squamous cell carcinomas of Latin American patients.

We compared the top altered genes in Latin Americans (all of them HPV-positive) with those described in three other populations (European, Asian, and North American) (in which most of the tumors are HPV-negative) (Supplementary Table S6). These populations present some commonly altered genes (Figure 3), of which only two, *NOTCH1* and *CASP8*, were found in the four populations. Latin Americans, North Americans, and Europeans share an alteration in *CDKN2A* and *FBXW7*, while Latin Americans, North Americans, and Asians have in common changes in *TP53*, *FAT1*, and *TTN* genes. The highest frequency of exclusive altered genes occurred in the Latin American population (88%), followed by North American (63%), European (60%), and Asian (18%). Among the exclusive genes in the Latin American population, we identified 10 genes related to cancer as novel altered genes potentially associated with HPV-associated PSCC: *KMT2C*, *SMARCA4*, *PTPRB*, *AJUBA*, *CR1* (in 20% of the tumors, each), *KMT2D*, *CIC*, *NBEA*, *FAM135B*, and *GTF2I* (in 15% of the tumors, each). Table 2 shows the variants detected in cancer-associated genes found exclusively in Latin Americans. Interestingly, all loci are in HPVis, according to HPVbase (http://crdd.osdd.net/servers/hpvbase/index.html (accessed on 17 March 2022)) [42].

### 3.4. Copy Number Variation in HPV-Associated PSCC

The CNV-seq analyses revealed 558 genes presenting ≥ 3 copies, of which 42 are associated with cancers. *NOTCH1*, *PTCH1*, *TSC1*, *NUP214*, *SET*, *ABL1*, *TNC*, *FANCC*, *PLAG1*, *MYC*, *RAD21*, and *NUMA1* were detected in 10% of the tumors. When comparing the CNV-seq data with our previous data on CNAs detected by aCGH [10], we found 80 genes in common presenting gene copy gain. Among them, five cancer-associated genes were confirmed by both analyses as the most frequent genes with gains: *NOTCH1*, *MYC*, *NUMA1*, *PLAG1*, and *RAD21*. Deletions were less frequent in both aCGH and CNV-seq analysis; however, we highlight that 30% of the tumors showed *SMARCA4* loss (Figure 2C). Table 3 presents the main altered cytobands and the most frequent genes with gain, all of them localized at HPVis for subtype 16.

### 3.5. UTRs Variants and Their Biological Consequences in miRNA-mRNA Interactions

Among all altered genes, 5.5% have variants in their UTRs (Figure 1). To investigate the biological consequences of these variants, we identified miRNAs that may regulate these genes, as well as their seed regions (miRNA × mRNA interacting regions) that may be impacted by the variants. As a result, 14 genes were found to be regulated by miRNAs (miRabel score ≤ 0.05), four of which are cancer-associated genes (*CARD11*, *CSMD3*, *KDR*, and *TLX3*). The variants are located within the gene hybridization sites (Supplementary Table S7). The *CSMD3* gene (regulated by miR-1237-3p, miR-224-5p, and miR-132-3p) presented the highest number of hybridization regions affected by the c.-19T > C variant (sites for miR-1237-5p). In addition, the ‘c.-19T > C’ variant has not yet been described in the dbSNP and COSMIC databases.

### 3.6. Spectrum of Somatic Variants in HPV-Associated PSCC

**Figure 4:**
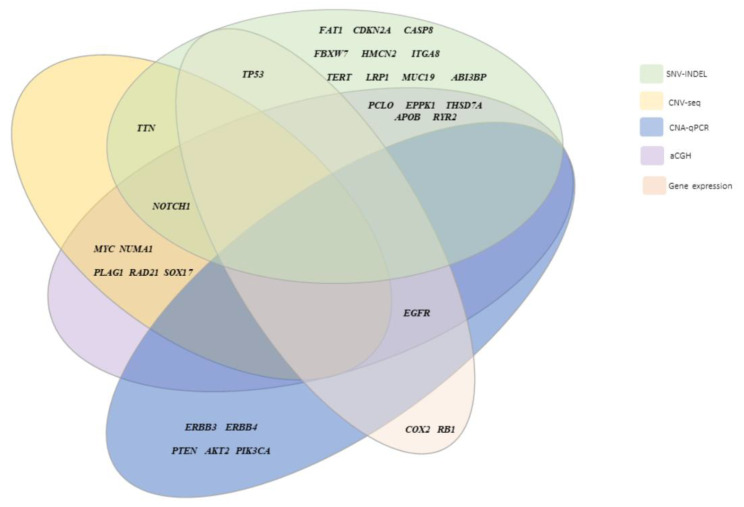
The spectrum of main genes exhibiting somatic variants in human papillomavirus-associated penile squamous cell carcinomas in Latin American patients.

Finally, to generate a spectrum of somatic variants in HPV-associated PSCC from Latin Americans, we crossed WES data with genomic alterations identified in our previous study [10], in which tumors were characterized for GEC and CNA. The profile of the most frequently altered genes in both studies is represented in Figure 4.

### 3.7. Molecular Pathways in HPV-Associated PSCC

**Figure 5:**
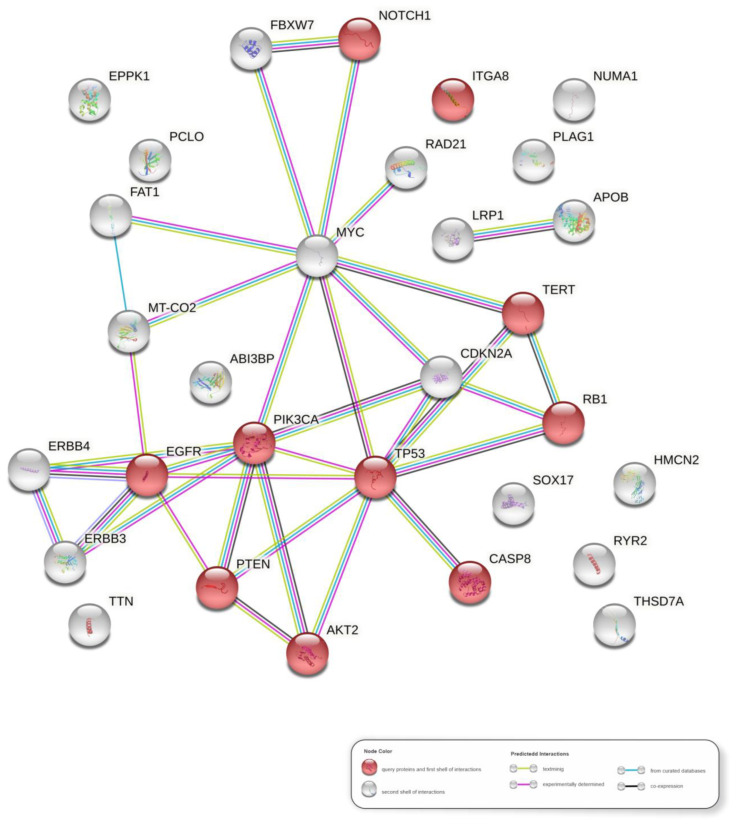
Protein–protein interaction network of the main genes presenting somatic variants in human papillomavirus-associated penile squamous cell carcinomas from Latin Americans. The red nodes represent genes supporting the human papillomavirus infection pathway.

We predicted the main pathways (highest confidence) by using the most frequently altered genes in the HPV-associated PSCC (Figure 4). Supplementary Table S8 lists the top 20 KEGG pathways identified and ranked by FDR value. The HPV infection pathway (hsa05165) is one of the top five, with *PIK3CA*, *RB1*, *TP53*, *EGFR*, *NOTCH1*, *TERT*, *CASP8*, *PTEN*, *ITGA8*, and *AKT2* supporting it. The network of gene interactions supporting the top pathways for HPV-associated PSCC in Latin Americans is visualized in Figure 5.

## 4. Discussion

Our study revealed the first mutational PSCC signature of Latin Americans, for whom we performed a high-throughput WES. Our study population is from the state of Maranhão in Northeast Brazil, where HPV prevalence has been reported to be among the highest in the world [5]. We also compared our findings with previous WES studies of penile tumors, in which mutational landscapes were described in Europeans [14], Asians [15], and North Americans [16], all of which had a similar sample size (30 ± 3.5). This comparison is particularly interesting because, in addition to the differences in genetic backgrounds and HPV status (mostly HPV-negative in the previous studies), the clinical and social characteristics of patients from developed countries contrast with those from Brazil.

HPV is the second most prevalent cancer-causing viral agent worldwide and is related to several types of tumors, such as cervical, oropharyngeal, and anogenital, including penile [44]. However, while HPV DNA is identified in over 90% of cervical carcinoma cells [7], there is substantial variation in viral positivity among penile tumors, mainly due to differences in molecular testing. Our study population is a subset from 55 previously investigated PSCC cases [10], with the highest HPV positivity among other PeCa populations [45]. This higher HPV positivity is likely a result of two more sensitive detection/genotyping methods (DNA sequencing in addition to nested-PCR able to detect 40 HPV subtypes), improving the accuracy compared to the use of a single method. As result, in this present study, 100% of the tumors were HPV positive, of which 96% were high risk.

The high-coverage WES enabled us to detect variants in exonic and intronic regions, splicing sites, UTR, and ncRNA, in addition to the major CNV-seq. Additionally, these alterations were integrated with CNAs and gene expression variations detected in 18/30 cases [10,11]. Among the altered genes found in at least 15% of tumors, 18 (*NOTCH*, *FAT1*, *TP53*, *CDKN2A*, *FBXW7*, *CASP8*, *SMARCA4*, *PTPRB*, *KMT2C*, *CR1*, *AJUBA*, *KMT2D*, *MTOR*, *NBEA*, *LRP1B*, *FAM135B*, *GTF2I*, and *CIC*) had already been linked to different types of cancer [38]. Considering the number of variants per gene size, *CDKN2A*, *TP53*, *CASP8*, and *FBXW7* were the hotspots. Schwaederle et al. [46] proposed *CDKN2A*, *NOTCH1*, *TP53*, and *FBXW7* as “squamousness” genes in head and neck, lung, cutaneous, gastrointestinal, and gynecologic cancers. Interestingly, all of those genes were altered at a high frequency in our cases and were classified as PSCC. Therefore, our data strengthen the importance of translational research that focuses on potential molecular targets that are common to squamous cell cancers, thereby mitigating the difficulties associated with conducting effective clinical trials due to the low prevalence of PeCa worldwide.

*CDKN2A* (p16) expression is a predictive marker for HPV infection [47], and as such, practically all HPV-associated cancers overexpress p16. HPV-unrelated tumors are often more aggressive and p16-negative, which is usually inactivated by homozygous deletion and promoter hypermethylation [48,49]. In the present study, *CDKN2A* had the highest frequency (35% of cases) of tumor-shared pathogenic variants, with all being stop-gain (p.R80X, p.R58X, and p.W110X). Previously, the nonsense variant was also reported, but in only two p16-negative penile tumors [16]. Similarly, other authors attributed loss-of-function variants to explain the negativity of p16 in 5% of HPV-associated cervical cancer [50]. Hence, considering the high frequency of *CDKN2A* nonsense variants in high-risk HPV advanced PSCC, we reinforce that p16 immunoexpression may be ineffective as an HPV marker in a significant subset of PeCa. Our results corroborate those of Zito Marino et al. [51], who highlighted the importance of using at least two methods to avoid the misclassification of HPV penile tumors in clinical practice.

By comparing our mutational signature to those reported before [14,15,16], we identified a common list of altered genes. Latin Americans’ signature shares gene alterations with North Americans and Europeans (*CDKN2A* and *FBXW7*) and with North Americans and Asians (*TP53*, *FAT1*, and *TTN*). These genes have been described as being involved in the establishment and progression of other tumors [52,53,54] and may also be potential drivers of this rare carcinoma. *NOTCH1* and *CASP8* were found to be frequently altered in all PeCa WES studies (LA = 33% and 17%; NA = 41% and 24%, respectively; EU =12.5% each; AS = 13.3% each). Feber’s group [14] proposed two mutational signatures based on the HPV status: an HPV-associated APOBEC mutation signature and an NpCpG mutation signature in HPV-negative disease, whereas Wang and collaborators [15] identified the Notch, RTK–RAS, and *hippo* pathways as the most disrupted pathways. Chahoud et al. [16] highlighted a molecular similarity between PSCC and head and neck squamous cell carcinoma (HNSC), specifically involving the Notch pathway, corroborating our findings of *NOTCH1* variants in 50% of cases, as well as those of Wang et al. [15].

It is worth noting that whole-genome sequencing was performed on a panel of HPV-negative and epithelial-like PSCC cell lines, in which the exomic variants were highly consistent with their corresponding cancer tissues [55]. Similar to our study, *TP53*, *CDKN2A*, and *NOTCH1* were among the most commonly altered genes. Furthermore, the authors observed amplification of *MYC*, *PLAG1*, and *EGFR*, revealing MAPK, Jak-STAT, TGF-β, Notch, and apoptosis signaling pathways as major players in the PeCa. Altogether, these results point out that SNV/Indels in these genes may be related to penile carcinogenesis regardless of HPV infection. On the other hand, we have previously suggested that downregulation of *TP53* and *RB1* mRNAs in PSCC [10] occurs through the upregulation of 13 miRNAs mapped at HPVis [12]. We also reported the impact of HPVis-localized CNAs on miRNA/mRNA interactions, which may alter critical pathways, such as P53 signaling, *hippo* and TGF-β signaling, proteoglycans in cancer, and viral carcinogenesis [11].

Although studies have described potential targets for PeCa treatment (see [45] for a review), most of them are frequently altered in other cancers; only a few clinical trials are available, with discouraging endpoints. Furthermore, despite the association between prognosis and HPV status [47], treatment is not chosen based on HPV infection. Thus, cisplatin-based chemotherapy remains the most used treatment, although there is no consensus on a standard regimen and, unfortunately, the majority of patients has a poor response to treatment [56].

We describe ten novel PSCC-associated genes, all of which have variants found exclusively in Latin Americans (*KMT2C*, *SMARCA4*, *PTPRB*, *AJUBA*, *CR1*, *KMT2D*, *NBEA*, *FAM135B*, *GTF2I*, and *CIC*). Notably, all of these genes are at HPVis, mainly for subtype 16, which is the most prevalent in our samples. HPV integration can cause cancer by activating oncogenes or inactivating tumor suppressors [42,43], and, although the process is still unclear, HPV infection has been related to penile carcinogenesis [7,57]. Four of the novel genes were described as predictive markers and potential pharmacological targets (https://www.dgidb.org (accessed on 3 May 2022) [58]); *SMARCA4* (Tazemetostat, Abemaciclib, Ribociclib, Vinorelbine, Palbociclib and Cisplatin), *CIC* (Vemurafenib, Selumetinib and Trametinib), *NBEA* (Metformin), and *CR1* (Eculizumab). 

In terms of targeted therapies, the mitogen-activated protein kinase (MAPK) pathway has received considerable attention; however, most MAPK inhibitors induce chemoresistance [59]. Furthermore, some of these inhibitors are highly selective, such as Vemurafenib, a drug with specificity for BRAF-V600E tumors [31], which were never reported in PeCa. On the other hand, although the *SMARCA*4 loss is not directly targetable, Januario and collaborators [60] demonstrated that inhibiting histone methyltransferase EZH2 was effective in *SMARCA4*-mutated cancer cell lines derived from various tumor types. Clinical trials have also demonstrated that Tazemetostat, an oral EZH2-specific inhibitor, has a favorable safety profile and antitumor activity [61,62,63,64]. Studies have also indicated that combining tumor immunotherapy with epigenetic modifiers, particularly EZH2, could enhance treatment efficacy (see Reference [65] for a review). Additionally, given the efficacy of CDK4/6 inhibitors in certain types of cancers harboring *SMARCA4* loss [66], we highlight that the deletion and nonsense variants in *CDKN2A* (p16) were found in 30% and 35% of our tumor samples, respectively. Hence, considering that p16 normally inhibits CDK4 and CDK6 [67], the use of tyrosine kinase inhibitors in tumors with *SMARCA4* and *CDKN2A* deficiency is a rational potential therapeutic option for this set of PSCCs.

We identified noncoding variants in the *TERT* promoter (*TERT*p) in 45% of tumors, with G250A (rs1561215364) and G228A (rs1242535815) accounting for 66.7% and 22.2%, respectively. Both G > A transitions were described as the most frequent in a large core of various cancers, 73% of which overexpressed TERT [68]. In contrast to a recent study [69] that linked *TERT*p alterations to non-HPV-related PSCC, we emphasize that all tumors with these alterations are HPV positive in the current study. *TERT* is normally silenced in nontumor cells but is reactivated via regulatory mechanisms that maintain the telomere length in human cancer cells, allowing them to survive. Interestingly, GABP (GA-binding protein) was identified as a critical transcription factor specifically recruited for *TERT* activation via the G > A mutant promoter [70]. Hence, the high frequency of these variants in PSCC provides opportunities for the development of anticancer strategies and applications of *TERT* as a potential biomarker for the diagnosis and prognosis of neoplasms [71]. Additionally, we identified an exonic p. (Val905Met) variant and a novel upstream (TERT:GG > AA at 1295127-1295128) variant, making *TERT* and *NOTCH1* the most frequently altered genes in HPV-associated PSCC (50% each).

The WES also allowed us to identify CNV-seq in 42 cancer-associated genes with cn > 3, including *NOTCH1*, *MYC*, *NUMA1*, *PLAG1*, and *RAD21* amplified in 10% of tumors. These findings confirm our previous aCGH data, showing that all alterations are mapped at high-risk HPVis. Moreover, we were able to identify genes presenting variants at miRNA binding sites (seed bindings in UTR regions). MiRNAs normally interact with the target gene/mRNA via their seed sequence (5′ end) or the untranslated region (3′UTR) of the gene [72]. Base pairing has also been reported in the 5′ UTR region and in the coding region [73]. Herein, we found 14 genes with UTR variants, of which four cancer-associated genes harbor variants in 3′ UTR (*KDR* and *CARD11*) and 5′ UTR (*CSMD3* and *TLX3*). These genes have been described as oncogenes, coding for vascular endothelial growth factor receptor 2 [57,74,75], promoting cell survival by activating NF-kb [76,77], activating downstream MAPK/ERK signaling, inhibiting p53 and activating cMyc [78,79], and promoting cell growth in hematological neoplasms by activating LINC00478/miR-125b [80]. The UTR variants can alter the binding site of miRNAs, impairing their regulatory capacity; they may also generate a new binding site for other miRNAs, resulting in an imbalance of cellular homeostasis. A limited number of studies have described variants in UTRs in cancer [81,82,83], and our findings form the basis for future studies into the biological and functional impact of somatic variants in these regions on penile carcinogenesis.

Finally, the spectrum of genomic alterations obtained across an integrative analysis of the copy number and gene expression alterations in the analyzed penile tumors allowed us to predict the main pathways involved in penile carcinogenesis. As previously discussed, although other authors have proposed a link between HPV infection and penile carcinogenesis, the variety of tissue collection methods and techniques used to detect the virus allowed false-negative results. Thus, the association of specific markers or cellular pathways with viral infection still remains controversial. We used two highly sensitive molecular methods for virus detection/genotyping, which allowed us to identify the virus precisely in the tumor samples. Nevertheless, our methods did not detect active infection, which was a limitation of our study, as well as the absence of HPV-negative cases.

The enrichment analysis revealed the HPV infection pathway (hsa05165) among the top five, supported by the interaction network of the genes *PIK3CA*, *RB1*, *TP53*, *EGFR*, *NOTCH1*, *TERT*, *CASP8*, *PTEN*, *ITGA8*, and *AKT2*. This corroborates with our previous study in which HPV-mediated viral carcinogenesis was also supported for both gene and miRNAs CNAs [11]. Furthermore, most of the altered genes identified by WES, presenting SNV/Indels or CNV-seq, as well as the majority of altered cytobands identified by aCGH, lie within HPVis, reinforcing the pivotal role of HPV infection in genomic alterations through integration of the virus into the host genome.

## 5. Conclusions

The data from the first whole-exome sequencing of penile tumors from Latin Americans that are characterized by HPV infection and an advanced disease revealed that most of the alterations are located at HPVis. *NOTCH1*, *TERT*, *TTN*, *FAT1*, *TP53*, *CDKN2A*, *RYR2*, *CASP8*, *FBXW7*, *HMCN2*, and *ITGA8* were the most frequently altered genes with SNV/Indels. In addition, ten novel genes (*KMT2C*, *SMARCA4*, *PTPRB*, *AJUBA*, *CR1*, *KMT2D*, *NBEA*, *FAM135B*, *GTF2I*, and *CIC*) were found in the Latin American population only, suggesting innovative targets with a potential therapeutic benefit for HPV-associated PSCC. We highlighted potential tyrosine kinase inhibitors since a significant proportion of tumors have *SMARCA4* and *CDKN2A* deficiency. In addition, *NOTCH1*, *MYC*, *NUMA1*, *PLAG1*, and *RAD21* were found to be amplified in 10% of tumors, thus supporting the aCGH findings. We also described, for the first time, UTRs variants (*KDR*, *CARD11*, *CSMD3*, and *TLX3*), impacting the miRNAs’ binding sites in penile tumors. All of these genomic alterations allowed us to predict important molecular pathways and biological markers involved in penile carcinogenesis, thereby improving our knowledge and confirming previous studies. The HPV molecular pathway was highly supported by our findings. Additionally, our data strengthen the importance of translational research that focuses on potential molecular targets common to squamous cell cancers, thereby mitigating the difficulties associated with conducting effective clinical trials due to the low prevalence of penile tumors worldwide. We further highlight the importance of collaborative research involving the integration of data from different populations, whose genetic, socioeconomic, and cultural characteristics, in addition to HPV status, must have a substantial impact on the initiation, development, and treatment response of penile cancer.

## Figures and Tables

**Table 1:** Clinical and histopathological profile of 30 PSCC patients analyzed by WES, qPCR (CNA and gene expression), and aCGH (n = 18).

Parameters	WES and qPCR (%)	aCGH (%)	Parameters	WES and qPCR (%)	aCGH (%)
Histologic site			Histologic type		
Glans	10 (33.3)	5 (27.8)	Usual	12 (40.0)	7 (38.8)
Glans + foreskin	8 (26.7)	8 (44.4)	Condylomatous	14 (46.6)	9 (50.0)
Foreskin	2 (6.7)	2 (11.1)	Mixed	2 (6.7)	1 (5.6)
Glans and other areas	10 (33.3)	3 (16.7)	Basaloid	2 (6.7)	1 (5.6)
Predominant lesion			Surgical procedure:		
Ulcerated Vegetative Ulcer/vegetative Verrucous Nodular ulcerated No information	11 (36.7) 10 (33.3) 3 (10.0) 2 (6.7) 3 (10.0) 1 (3.3)	6 (33.3) 6 (33.3) 2 (11.1) 2 (11.1) 2 (11.1) 0	Preserved penis Amputation (partial) Amputation (total) Glandectomy (total) No information	1 (3.3) 17 (56.7) 9 (30.0) 2 (6.7) 1 (3.3)	1 (5.6) 12 (66.6) 5 (27.8) 0 (0.0) 0 (0.0)
Lymphatic invasion			Perineural invasion		
Yes	5 (16.7)	3 (16.7)	Yes	9 (30.0)	5 (27.8)
No	22 (73.3)	13 (72.2)	No	18 (60.0)	11 (61.1)
No information	3 (10.0)	2 (11.1)	No information	3 (10.0)	2 (11.1)
Tumor Grade:			Tumor size (mm):		
G1	2 (6.7)	1 (5.6)	0.6–2.0	2 (6.7)	0
G2	20 (66.7)	12 (66.6)	2.1–5.0	17 (56.6)	13 (72.2)
G3	8 (26.6)	5 (27.8)	5.1–10.0	11 (36.7)	5 (27.8)
Primary tumor (T)			Infection HPV		
pT1 pT2 pT3 No information	5 (16.7) 11 (36.7) 13 (43.3) 1 (3.33)	3 (16.7) 7 (38.9) 8 (44.4) 0	Positive Negative	30 (100.0) 0	18 (100.0) 0
Phimosis			Marital status		
Yes	15 (50.0)	7 (38.9)	Married	20 (66.7)	15 (83.3)
No	9 (30.0)	6 (33.3)	Single	7 (23.3)	3 (16.7)
No information	6 (20.0)	5 (27.8)	No information	3 (10.0)	0

PSCC, penile squamous cell carcinoma; CNA, copy number alteration; aCGH, array Comparative Genomic Hybridization; WES, whole-exome sequencing.

**Table 2:** Cancer-associated genes presenting variants detected by whole-exome sequencing in human-papillomavirus—associated penile squamous cell carcinomas exclusively altered in Latin American patients.

Gene	Cytoband	Integration Sites (HPV Subtypes) *	Variants			
*KMT2C*	7q36.1	yes (16, 18)	c.11261C > T	c.4844G > A	c.162-17T > G	c.9059C > G
*KMT2D*	12q13.12	yes (16, 18)	c.511C > T c.9614G > C	c.9232G > C c.9186G > C	c.8914G > A c.12133C > T	
*SMARCA4*	19p13.2	yes (16, 18)	c.3575G > A	c.1249C > T	c.3694G > A	chr19:g.11003471C > T
*CIC*	19q13.2	yes (16, 18)	c.1264C > T	chr19:g.42274618G > A	chr19:g.42287248CC > TT:p.?	
*PTPRB*	12q15	yes (18)	c.2886G > C	c.979G > C	c.2357C > G	chr12:g.70609388C > T
*NBEA*	13q13.3	yes (16, 26) **	c.6226C > T c.1572T > G	c.4644 + 5G > A	c.6060G > A	c.5463C > A
*FAM135B*	8q24.23	yes (16, 18, 45)	c.2974T > A	chr8:g.138132825A > C		
*GTF2I*	7q11.23	yes (16)	c.178G > C	c.20C > G	c.233A > G	chr7:g.74589997C > A
*AJUBA*	14q11.2	yes (16, 18)	c.1006G > C	chr14:g. 22978448GAGAA > -:p.?	c.762_ 763insGA	c.445dupG
*CR1*	1q32.2	yes (16, 18)	c.4067G > C	c.68G > A	c.5383G > T	chr1:g.207588649A > T

* Data were obtained from HPV base (http://crdd.osdd.net/servers/hpvbase/index.html, last accessed on 17 March 2022) [42]; ** Data were obtained from Virus Integration Site DataBase (VISDB) (https://bioinfo.uth.edu/VISDB/index.php/homepage, last accessed on 17 March 2022) [43].

**Table 3:** Cancer-related genes with gain (≥3 copies) detected in 10% of human-papillomavirus-associated penile squamous cell carcinomas from Latin American patients. The specified cytobands have over 10 Kb.

Gene	Start	End	Cytoband	HPV Integration Site (Subtypes) *
*NOTCH1* ^ ☨^	89311043	138219684	9q22.2–9q34.2	yes (16, 18)
	136407694	136518542	9q34.3–9q34.3	yes (16, 18)
*PTCH1*	89311043	138219684	9q22.2–9q34.2	yes (16, 18)
	84866993	98578322	9q21.33–9q22.31	yes (16, 18)
*TSC1*	89311043	138219684	9q22.2–9q34.2	yes (16, 18)
	131060785	133209449	9q34.12–9q34.2	yes (16, 18)
*NUP214*	89311043	138219684	9q22.2–9q34.2	yes (16, 18)
	131060785	133209449	9q34.12–9q34.2	yes (16, 18)
*SET*	89311043	138219684	9q22.2–9q34.2	yes (16, 18)
	128220134	128822687	9q34.11–9q34.11	yes (16, 18)
*ABL1*	129873232	131057397	9q34.11–9q34.12	yes (16, 18)
	89311043	138219684	9q22.2–9q34.2	yes (16, 18)
*TNC* ^ ☨^	89311043	138219684	9q22.2–9q34.2	yes (16, 18)
	114792157	120990030	9q32–9q33.1	yes (16, 18)
*FANCC* ^ ☨^	89311043	138219684	9q22.2–9q34.2	yes (16, 18)
	84866993	98578322	9q21.33–9q22.31	yes (16, 18)
*PLAG1* ^ ☨^	54136261	89971540	8q21.12–8q13.2	yes (16, 18, 68)
	56072840	143934540	8q21.12–8q24.22	yes (16, 18, 68)
*MYC* ^ ☨^	117528401	133257113	8q24.12–8q24.22	yes (16, 18)
	56072840	143934540	8q21.12–8q24.22	yes (16, 18, 68)
*RAD21*	109399826	117521303	8q23.2–8q24.11	yes (16, 18)
	56072840	143934540	8q21.12–8q24.22	yes (16, 18, 68)
*NUMA1*	70078357	73083990	11q13.3–11q13.4	yes (16)
	71463803	72023397	11q13.4–11q13.4	yes (16)

^☨^ HPV target gene; * data obtained from HPVbase (http://crdd.osdd.net/servers/hpvbase/index.html (accessed on 17 March 2022)) [42] and Virus Integration Site DataBase [43].

## Data Availability

The data presented in this study are available upon request from the corresponding author. The data are not publicly available due to patient confidentiality.

## Supplementary Materials

The following supporting information can be downloaded at: https://www.mdpi.com/article/10.3390/cancers14143514/s1. Figure S1: Flowchart representing the study design. Fresh penile squamous cell carcinomas were collected from 30 Latin American patients. Tumors were screened for human papillomavirus (HPV) infection and were genotyped in most cases (5 cases yielded insufficient DNA). Whole-exome sequencing (WES) was performed in 20 paired tumors and adjacent normal tissues and 10 single tumors. Sequencing results were used for identifying somatic alterations. Furthermore, enrichment for miRNA–mRNA interactive regions (miR-mRNAir) and HPV integration sites (HPVis) was performed. The copy number variants (CNVs) were contrasted to previous CNA results of the same cases based on aCGH, with validation of selected genes by qPCR and RT-qPCR^10^. The mutated genes identified in this study were compared to those described by three previous studies reporting WES data from PeCa patients from Europe^14^, Asia^15^, and North America^16^. Figure S2: Depth and coverage of (A) HPV-associated penile squamous cell carcinoma samples (n = 30) and (B) nontumor adjacent penile tissues (n = 20). Table S1: Clinical–histopathological characterization of the Latin Americans’ patients with HPV-associated penile squamous cell carcinomas (n = 30). Table S2: Quality control of the whole-exome sequencing performed in 30 HPV-associated penile squamous cell carcinomas. Table S3: Cancer-associated genes presenting alterations in HPV-associated penile squamous cell carcinomas from Latin Americans. Table S4: Top 84 genes with somatic variants identified by WES in HPV-associated penile squamous cell carcinomas from Latin Americans, and the normalized number of variants based on gene size (bp). Cancer-associated genes are highlighted in bold. Table S5: Variants detected in the most altered genes in Latin Americans with HPV-associated penile squamous cell carcinomas. Table S6: Main altered genes described in three previous studies in which whole-exome sequencing was performed in penile squamous cell carcinomas from Europeans [14], Asians [15], and North Americans [16], compared to Latin Americans (this study). Table S7: Genes with variants in untranslated regions (UTRs), and the miRNAs that binding by seed region. Table S8: The top twenty KEGG pathways identified from a spectrum of major genes with somatic variants in HPV-associated penile squamous cell carcinomas from Latin Americans, ranked by FDR value.
