# Supplementary material for: Mutational Signature and Integrative Genomic Analysis of Human Papillomavirus-Associated Penile Squamous Cell Carcinomas from Latin American Patients
Source: Cancers (Basel). 2022 Jul 20;14(14):3514. doi: 10.3390/cancers14143514 (PMC9316960; doi:10.3390/cancers14143514)
Supplement: Supplementary file 1 [file cancers-14-03514-s001.zip › cancers-1775552-supplementary.pdf]

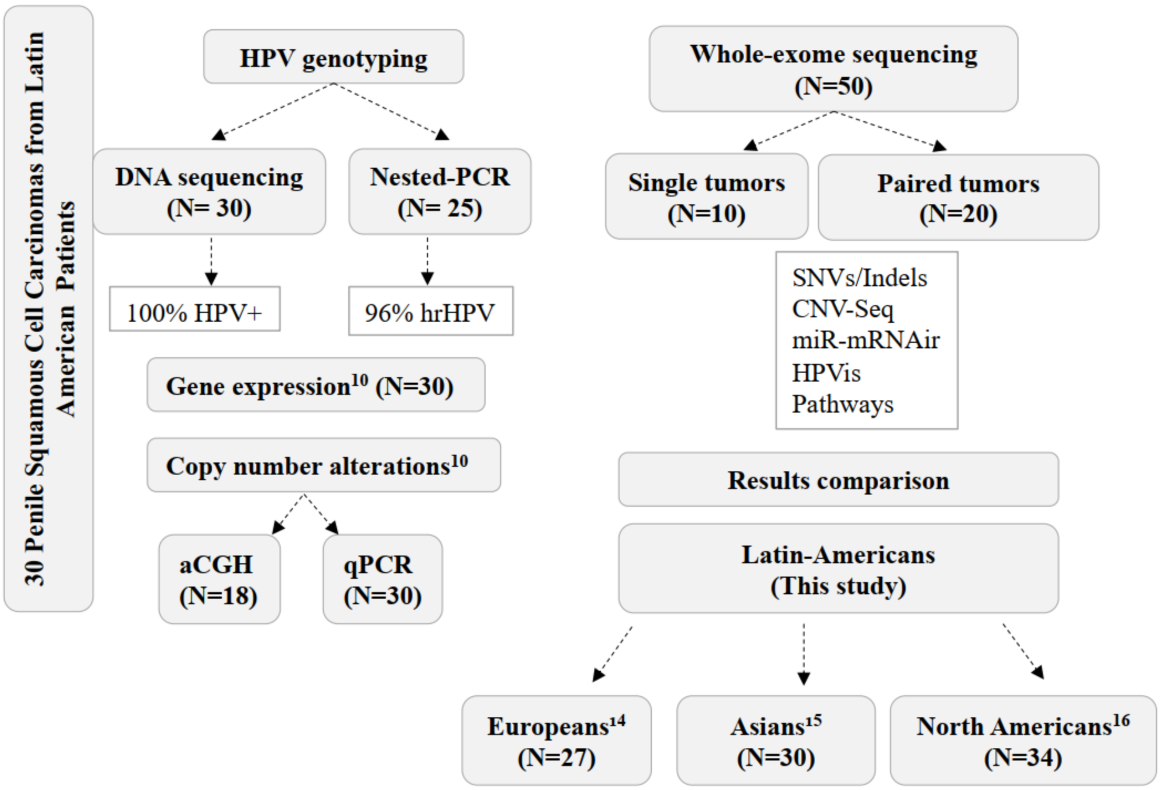

Figure S1. Flow chart representing the study design. Fresh penile squamous cell carcinomas were collected from 30. Latin American patients. Tumors were screened for Human Papillomavirus (HPV) infection, which were genotyped in most cases (5 cases yielded insufficient DNA). Whole-exome sequencing (WES) was performed in 20 paired tumors and adjacent normal tissues and 10 single tumors. Sequencing results were used for identifying somatic alterations in PSCCs. Further, enrichment of miRNA-mRNA interactive regions (miR-mRNAir) and HPV integration sites (HPVis) was performed. The copy number variants (CNV) were contrasted to previous CNA results of the same cases based on aCGH, with validation of selected genes by qPCR and RT-qPCR [10]. The mutated genes identified in this study were compared to those described by three previous studies reporting WES data from PeCa patients from Europe [14], Asia [15], and North America [16].

A

B

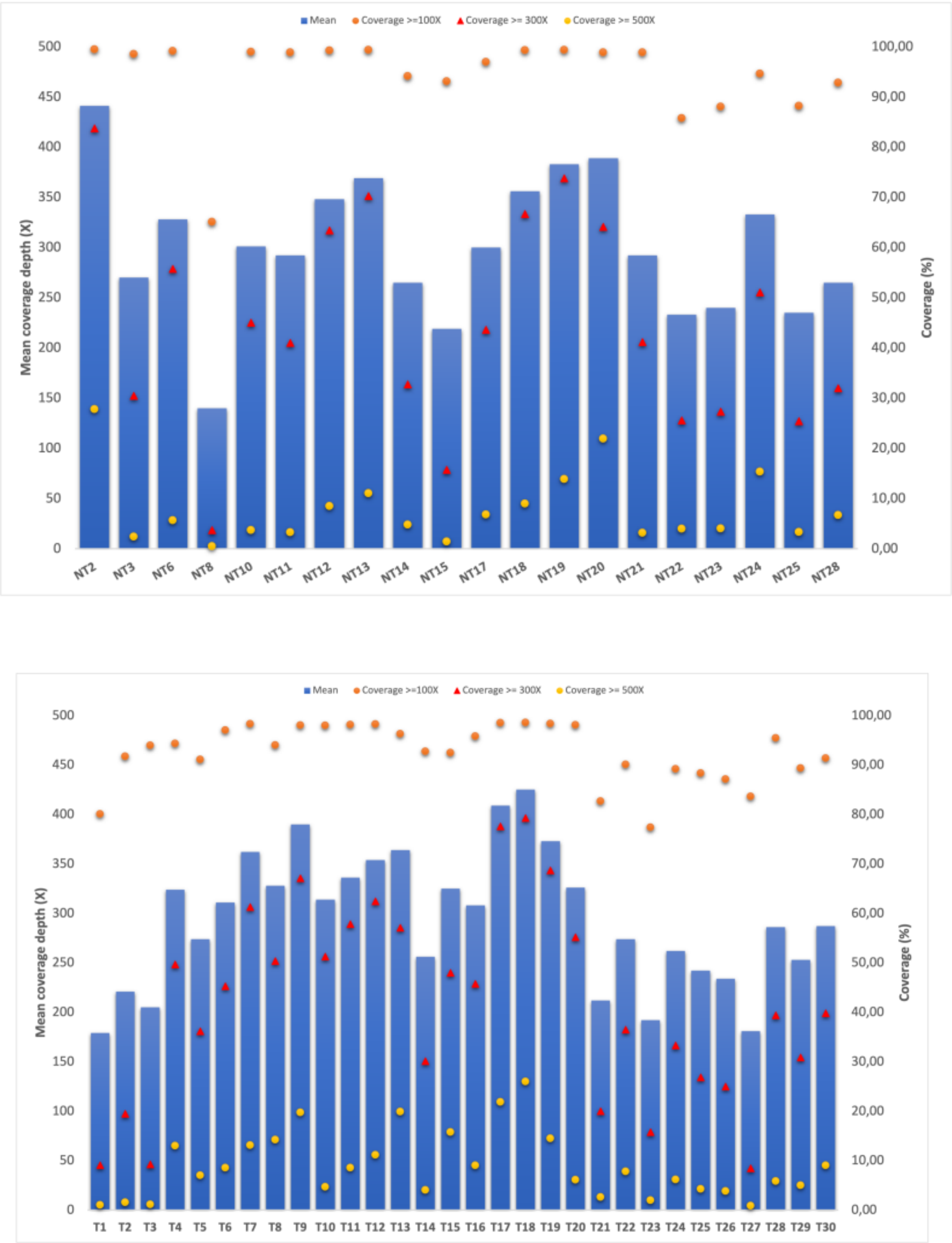

Figure S2. Depth and coverage of (A) HPV-associated penile squamous cell carcinoma samples (n = 30) and (B) nontumor adjacent penile tissues (n = 20).

Table S1. Clinical-histopathological characterization of the Latin Americans' patients with HPV-associated Penile Squamous Cell Carcinomas (n=30).

Case.	Age	Marital status	Phymosis ocurrence	Smoking	Alcoholism	HPV subtype	Histologic type	Predominant lesion	Tu. size [b]	Tu. grade	Tu. stage	Lymphatic invasion	Perineural invasion
T1	45	N/A	yes	N/A	N/A	positive [a]	usual	ulcerated	5	G1	pT2	no	yes
T2	62	married	no	N/A	N/A	16	usual	ulcerated	2.5	G2	pT3	yes	yes
T3	80	married	N/A	yes	N/A	16	condilomatous	ulcerated	10	G2	pT2	no	no
T4	80	single	yes	yes	yes	16	usual	ulcerated	1.3	G2	pT1	no	no

T5	81	married	yes	yes	N/A	18,53	condilomatous	ulcerated	2.1	G2	pT1	no	no
T6	76	N/A	yes	N/A	N/A	30	usual	ulcerated	10	G2	PT3	yes	yes
T7	37	married	yes	no	yes	positive [a]	usual	nodular ulcerated	5.0	G3	pT3	no	yes
T8	54	married	yes	yes	yes	positive [a]	usual	vegetative	0.8	G2	pT2	yes	no
T9	78	married	N/A	yes	yes	16,06	usual and basaloid	vegetative	4	G3	pT1b	no	no
T10	52	married	yes	no	no	16	usual	ulcerated	1.2	G2	pT1	no	no
T11	85	single	no	N/A	N/A	16	condilomatous	nodular ulcerated	4	G3	pT3	no	yes
T12	83	married	no	yes	yes	16	usual	ulcerated	4.5	G3	pT3	no	yes
T13	51	married	yes	no	no	16	basaloid	vegetative	N/A	G2	pT2	N/A	N/A
T14	40	married	no	no	no	59,66	condilomatous	ulcer-vegetative	3	G2	pT2	no	no
T15	43	single	yes	yes	yes	16	usual	ulcerated	9	G3	pT3	no	yes
T16	68	married	N/A	no	no	16,66	condilomatous	verrucous	5.5	G1	pT3	no	no
T17	41	married	yes	yes	yes	56	condilomatous	vegetative	3.5	G2	pT2	no	no
T18	65	married	yes	yes	no	16	usual and basaloid	nodular ulcerated	N/A	G3	pT3	N/A	N/A
T19	34	single	yes	no	yes	56	basaloid	ulcer-vegetative	6	G2	pT3	no	no
T20	74	married	no	yes	yes	11,35,59	condilomatous	vegetative	9	G2	pT3	no	no
T21	65	married	N/A	N/A	N/A	16,74	condilomatous	vegetative	3.5	G2	pT2	no	no
T22	43	married	no	no	no	16	condilomatous	vegetative	2.5	G2	pT2	no	no
T23	51	single	N/A	yes	yes	16,35,59	usual	ulcer-vegetative	3	G2	pT2	yes	no
T24	64	married	no	yes	no	16	condilomatous	vegetative	6	G1	pT2	yes	yes
T25	31	single	yes	no	N/A	16	condilomatous	vegetative	5.5	G3	pT3	no	no
T26	71	married	no	no	no	16	usual	ulcerated	3.2	G2	pT3	no	no
T27	73	married	yes	yes	no	16	usual	ulcerated	N/A	G2	pt1	N/A	N/A
T28	67	married	N/A	no	no	16	condilomatous	vegetative	7	G2	pT2	no	no
T29	59	single	yes	no	yes	positive [a]	condilomatous	verrucous	5.5	G2	pT3	no	yes
T30	N/A	N/A	no	N/A	N/A	positive [a]	condilomatous	N/A	2.5	G2	N/A	no	no

PSCC: Penile Squamous Cell Carcinoma; [a] no information on HPV genotype; [b] Tu = tumor (mm); N/A = not available.

Table S2. Quality control of the whole-exome sequencing performed in 30 HPV-associated Penile Squamous Cell Carcinomas.

Sample	Paired-reads (million)	Paired-reads on-target (million)	DNA mapping (%)	Read Deapth	Coverage ≥100X	Q30
Tumor tissues						
**T1**	47.10	46.83	99.42	179	80.10	96.52%
**T2**	57.30	57.20	99.83	221	91.75	96.98%
**T3**	51.80	51.63	99.66	205	93.93	97.03%
**T4**	85.30	85.03	99.68	324	94.34	97.51%
**T5**	67.00	66.82	99.73	274	91.07	97.52%
**T6**	76.30	76.19	99.85	311	97.04	96.72%
**T7**	94.00	93.84	99.83	362	98.29	97.43%
**T8**	83.30	83.04	99.69	328	93.99	97.44%
**T9**	98.30	98.21	99.91	390	98.03	97.48%
**T10**	78.20	78.06	99.82	314	97.99	97.23%
**T11**	84.80	84.58	99.74	336	98.12	97.55%
**T12**	86.20	86.12	99.90	354	98.25	97.40%
**T13**	87.30	87.16	99.84	364	96.29	97.05%

**T14**	62.50	62.29	99.67	256	92.73	97.47%
**T15**	83.80	83.59	99.75	325	92.46	94.89%
**T16**	75.80	75.57	99.70	308	95.83	96.67%
**T17**	106.30	105.19	98.95	409	98.53	97.10%
**T18**	107.80	107.44	99.66	425	98.57	96.56%
**T19**	92.90	92.75	99.84	373	98.37	96.78%
**T20**	82.80	82.51	99.65	326	98.07	96.88%
**T21**	53.10	52.87	99.57	212	82.68	96.39%
**T22**	68.60	68.34	99.63	274	90.07	96.63%
**T23**	46.70	46.41	99.39	192	77.39	97.31%
**T24**	65.10	64.85	99.61	262	89.20	96.90%
**T25**	60.60	60.51	99.85	242	88.32	96.71%
**T26**	59.20	58.99	99.65	234	87.11	97.63%
**T27**	43.10	42.85	99.42	181	83.64	97.52%
**T28**	69.60	69.45	99.78	286	95.45	97.13%
**T29**	64.80	64.63	99.73	253	89.36	96.96%
**T30**	73.80	73.62	99.75	287	91.37	97.02%
**Mean**	**73.78**	**73.55**	**99.68**	**293.5**	**92.61**	**97.01%**
**SD**	**17.43**	**17.38**	**0.19**	**67.8**	**5.85**	**0.53**
**Non-tumor tissues**						
**NT2**	111.8	111.39	99.63	441	99.44	97.85%
**NT3**	69.7	69.33	99.46	270	98.52	96.63%
**NT6**	83.1	83.03	99.92	328	99.12	97.43%
**NT8**	34.2	34.09	99.68	140	65.09	97.25%
**NT10**	79.9	79.77	99.84	301	98.94	97.30%
**NT11**	73.5	73.36	99.81	292	98.87	97.27%
**NT12**	85.5	85.31	99.78	348	99.22	96.96%
**NT13**	94.7	94.52	99.81	369	99.34	96.83%
**NT14**	63.5	63.31	99.71	265	94.11	97.68%
**NT15**	54.7	54.48	99.59	219	93.09	96.46%
**NT17**	71.5	71.44	99.91	300	96.97	95.02%
**NT18**	91.9	91.74	99.83	356	99.28	96.81%
**NT19**	98.2	97.55	99.34	383	99.33	96.23%
**NT20**	95.8	95.62	99.81	389	98.86	95.52%
**NT21**	75.7	75.44	99.66	292	98.85	96.42%
**NT22**	59.3	59.00	99.49	233	85.77	96.10%
**NT23**	59.7	59.45	99.58	240	88.03	96.34%
**NT24**	80.7	80.37	99.59	333	94.59	96.22%
**NT25**	60.3	60.10	99.67	235	88.20	96.77%
**NT28**	63.4	63.06	99.47	265	92.81	96.05%
**Mean**	**75.36**	**75.12**	**99.68**	**299.95**	**94.42**	**96.65**
**SD**	**18.23**	**18.18**	**0.16**	**70.60**	**8.15**	**0.71**

Table 3. Cancer-associated genes presenting alterations in HPV-associated Penile Squamous Cell Carcinomas from Latin Americans.

**Genes**					
ABL2	*CDH17*	*ELF3*	*KMT2D*	*NUTM2A*	*SF3B1*
AKT1	*CDKN2A*	*ELF4*	*KRAS*	*OLIG2*	*SFRP4*
ANK1	*CDX2*	*EPHA7*	*LCP1*	*PBRM1*	*SH2B3*
ARHGAP5	*CEBPA*	*ERBB2*	*LMO2*	*PCM1*	*SKI*
ARID1B	*CIC*	*ERBB4*	*LRP1B*	*PDGFRA*	*SLC34A2*
ARID2	*CLTC*	*ETV1*	*MACC1*	*PICALM*	*SMARCA4*
ATM	*CLTCL1*	*ETV6*	*MAP2K1*	*PIK3CA*	*SOS1*
BAP1	*CNBD1*	*EZR*	*MAP3K13*	*POLE*	*SPECC1*
BCL11B	*CNTNAP2*	*FAM135B*	*MAX*	*POLG*	*SPEN*
BCL3	*COL1A1*	*FAM47C*	*MDM2*	*POLQ*	*TBX3*
BCL6	*COL2A1*	*FAT1*	*MPL*	*PRCC*	*TLX3*
BCL7A	*CREB3L2*	*FAT3*	*MSN*	*PREX2*	*TOP1*

BCL9	*CRELD2*	*FBLN2*	*MTOR*	*PRKCB*	*TP53*
BCL9L	*CRTC3*	*FBXW7*	*MUC16*	*PTCH1*	*TP63*
BCR	*CSF1R*	*FES*	*MYCN*	*PTPN6*	*TRRAP*
BIRC6	*CSMD3*	*FGFR3*	*MYH9*	*PTPRB*	*TSC2*
BRAF	*CTCF*	*FLI1*	*MYOD1*	*PTPRC*	*TUB*
BRCA1	*CTNNB1*	*FLT4*	*NBEA*	*PTPRD*	*UBXN11*
BRCA2	*CTNND2*	*FUS*	*NCOA1*	*PTPRT*	*WNT8B*
BRD3	*CUX1*	*GNAS*	*NCOA2*	*RB1*	*ZEB1*
BRD4	*CYLD*	*GPC5*	*NCOA4*	*RBM39*	*ZNF479*
CACNA1D	*DCC*	*HERPUD1*	*NCOR2*	*RGPD3*	
CARD11	*DDR2*	*HNRNPA2B1*	*NF1*	*RHPN2*	
CASP8	*DDX5*	*HOOK3*	*NFE2L2*	*RNF213*	
CBL	*DDX6*	*ISX*	*NONO*	*RPL22*	
CBLB	*DNM2*	*JAK3*	*NOTCH1*	*RSPO3*	
CBLC	*DNMT3A*	*KDM6A*	*NOTCH2*	*RUNX1*	
CCND3	*DROSHA*	*KDR*	*NT5C2*	*RUNX1T1*	
CD79A	*ECT2L*	*KIAA1549*	*NTRK3*	*SETBP1*	
CDC73	*EGFR*	*KMT2C*	*NUP214*	*SETD1B*	

Table 4. Top 84 genes with somatic variants identified by WES in HPV-associated Penile Squamous Cell Carcinomas from Latin Americans, and the normalized number of variants based on gene size (bp). Cancer-associated genes are highlighted in bold.

Gene	Number of Tumors (%)	Number of variants	Gene size (bp)	Normalized number of variants (1000bp)
NOTCH1	10 (50)	10	13902	0.72
TTN	9 (45)	13	169889	0.08
TERT	9 (45)	10	7064	1.42
FAT1	9 (45)	10	18810	0.53
TP53	8 (40)	8	3097	2.58
CDKN2A	7 (35)	7	1912	3.66
RYR2	6 (30)	7	35904	0.19
LRP1	6 (30)	6	29302	0.20
HMCN2	5 (25)	8	33970	0.24
FBXW7	5 (25)	6	5162	1.16
CASP8	5 (25)	6	3634	1.65
MUC19	5 (25)	5	56064	0.09
ITGA8	5 (25)	5	9192	0.54
ABI3BP	5 (25)	5	18352	0.27
USH2A	4 (20)	5	29750	0.17
TRPM2	4 (20)	4	11262	0.36
SMARCA4	4 (20)	4	11901	0.34
PTPRB	4 (20)	4	13497	0.30
PCLO	4 (20)	4	20446	0.20
MYO15A	4 (20)	4	22443	0.18
KMT2C	4 (20)	4	26273	0.15
CR1	4 (20)	4	16322	0.25
CTNND2	4 (20)	4	8078	0.50
CASKIN1	4 (20)	4	7259	0.55
AJUBA	4 (20)	4	3330	1.20
KMT2D	3 (15)	6	25008	0.24
RYR3	3 (15)	5	35274	0.14
EPPK1	3 (15)	5	15467	0.32
CCDC168	3 (15)	5	21861	0.23
XIRP2	3 (15)	4	14495	0.28
VPS41	3 (15)	4	8212	0.49
SYNE1	3 (15)	4	55897	0.07
TMEM132C	3 (15)	4	5127	0.78

PPP2R3A	3 (15)	4	6385	0.63
OBSCN	3 (15)	4	49934	0.08
KRT6A	3 (15)	4	3290	1.22
CAPN9	3 (15)	4	6036	0.66
ZAN	3 (15)	3	17257	0.17
ZNF831	3 (15)	3	6034	0.50
ZZEF1	3 (15)	3	19541	0.15
SSPOP	3 (15)	3	32660	0.09
THSD7A	3 (15)	3	10574	0.28
SEL1L2	3 (15)	3	5219	0.57
SEMA6D	3 (15)	3	6383	0.47
TFAP2A	3 (15)	3	3174	0.95
RRP12	3 (15)	3	10206	0.29
PKHD1	3 (15)	3	25561	0.12
PLCE1	3 (15)	3	13366	0.22
RTN1	3 (15)	3	4280	0.70
PRDM9	3 (15)	3	4685	0.64
MTOR	3 (15)	3	18766	0.16
NEB	3 (15)	3	59824	0.05
MCF2L	3 (15)	3	10014	0.30
MROH2A	3 (15)	3	13000	0.23
PADI1	3 (15)	3	5046	0.59
MUC5AC	3 (15)	3	25566	0.12
NBEA	3 (15)	3	20327	0.15
LLGL1	3 (15)	3	6773	0.44
LRP1B	3 (15)	3	31847	0.09
HUWE1	3 (15)	3	28721	0.10
HCN1	3 (15)	3	4273	0.70
GUCY2D	3 (15)	3	6408	0.47
FRAS1	3 (15)	3	26897	0.11
FAM135B	3 (15)	3	8021	0.37
GFPT2	3 (15)	3	5750	0.52
GTF2I	3 (15)	3	9554	0.31
CIC	3 (15)	3	10499	0.29
CHD5	3 (15)	3	13467	0.22
CEP162	3 (15)	3	9412	0.32
CSMD2	3 (15)	3	25423	0.12
DMXL2	3 (15)	3	17510	0.17
CNTNAP5	3 (15)	3	8698	0.34
DNAH11	3 (15)	3	29500	0.10
COL11A1	3 (15)	3	18494	0.16
COL6A1	3 (15)	3	9304	0.32
ARHGEF15	3 (15)	3	4801	0.62
ADAMTS12	3 (15)	3	9650	0.31
ARHGAP31	3 (15)	3	6735	0.45
ADGRV1	3 (15)	3	36634	0.08
ATP10A	3 (15)	3	8419	0.36
AFF2	3 (15)	3	8387	0.36
CACNA1C	3 (15)	3	17453	0.17
ANK2	3 (15)	3	23304	0.13
APOB	3 (15)	3	19221	0.16

Table S5. Variants detected in the most altered genes in Latin Americans with HPV-associated Penile Squamous Cell Carcinomas.

Gene	HGVS id	Variants found in COSMIC Database	dbSNP	Variants not found in COSMIC Database	dbSNP
NOTCH1	NM_017617.5	c.1172C>T	-	c.1445A>C	-
		c.4015-6G>A	-	c.4389C>A	-
		c.1270G>A	rs1341448592	c.5613C>A	-
		c.1379C>T	rs776258761	c.3184_3185insTGTGCACT	-
				c.3439G>A	rs1589060737
				c.76C>T	rs1217905346
FAT1	NM_005245.4	c.2173C>T	-	c.3643-2A>G	-
		c.7789C>T	rs777631605	c.1550dupG	-
				c.10377delG	-
				c.4183+1G>C	-
				c.10830_10836del	-
				c.859_866del	-
				c.2879G>C	-
				c.5029delA	-
TTN	NM_001267550.2	c.77902G>A	rs756933390	c.21961+1G>A	-
				c.43498C>T	-
				c.107581G>A	-
				c.21115+6T>G	-
				c.94913C>A	-
				c.25230T>G	-
				c.23076C>A	-
				c.44929G>T	-
				c.25622C>T	-
				TTN:chr2:g.178652606G>C	-
				c.72331G>C	rs369671334
				c.45408G>T	rs72677225
TP53	NM_000546.6	c.529_546del	-		
		c.832_833delinsTT	-		
		c.722C>T	rs28934573		
		c.615T>A	rs786202222		
		c.743G>A	rs11540652		
		c.375+5G>A	rs1555526466		
		c.832C>T	rs17849781		
		c.574C>T	rs866380588		
		**c.733G>T	-		
		**c.817C>T	-		
		**c.818G>T	rs28934576		
CDKN2A	NM_000077.5	c.330G>A	rs121913389		
		c.238C>T	rs121913388		
		c.172C>T	rs121913387		
		c.329G>A	rs1057519852		
RYR2	NM_000077.5	c.4990G>A	rs749434532	c.11240G>C	-
				c.1572G>T	-
				c.10904_10916del	-
				c.4978C>T	-
				c.1613-3C>A	-
				c.7643T>A	-
				**RYR3:chr15:g.33581455AC>GT	-
CASP8	NM_001228.4			c.621C>G	-
				c.1170dupT	-
				c.948delC	-
				c.541A>G	-
				c.1321T>C	-

				c.1276_1277del	rs587776665
				**c.202C>T	rs777784105
FBXW7	NM_001013415.2			c.607T>C	-
				c.1273A>G	-
				c.1081_1082insA	-
				FBXW7:chr4:g.152328394AAA>-:p.?	-
				c.1159C>A	rs149680468
				**c.1082G>A	rs866987936
HMCN2	NM_001291815.2			c.8947G>A	-
				c.14834G>A	-
				c.5678-8C>T	-
				HMCN2:chr9:g.130349742G>A	-
				c.2920+17C>T	rs1033947767
				c.1798G>A	rs1363437156
				c.13840G>A	rs779508163
				c.2350C>T	rs564316104
ITGA8	NM_003638.3	c.2818G>A	rs369955410	c.2478+12C>G	-
				c.2672G>A	-
				c.652G>A	rs139218863
				c.209+11C>T	rs771423381
**CR1*	NM_000651.6	c.5383G>T	-	c.4067G>C	-
				CR1:chr1:g.207588649A>T	-
				c.68G>A	-
				**CR1:chr1:g.207618037C>G	-
USH2A	NM_206933.4	c.5329C>T	rs770329105	c.5168-19C>A	-
		c.3221G>T	-	c.6743A>G	-
				c.272G>T	-
PCLO	NM_014510.3	c.4150G>A	-	c.13171C>A	-
		c.4090G>A	-	c.14174A>G	rs768440811
TRPM2	NM_003307.4			c.1677C>G	-
				c.1391G>A	rs762016604
				c.2882G>A	rs752512943
				TRPM2:chr21:g.44382683C>T	rs1408103898
**SMARCA4*	NM_001128849.3	c.3575G>A	-	SMARCA4:chr19:g.11003471C>T	rs539548654
		c.1249C>T	-	**c.3573C>A	-
		c.3694G>A	-		
**KMT2C*	NM_170606.3	c.2872G>C	rs758966724	c.11261C>T	-
				c.4844G>A	-
				c.9059C>G	-
				c.162-17T>G	rs187902675
**AJUBA*	NM_032876.6			c.1006G>C	-
				c.762_763insGA	-
				c.445dupG	-
				AJUBA:chr14:g.22978448GAGAA>-:p.?	rs755876832
**PTPRB*	NM_001109754.4			c.979G>C	-
				c.2357C>G	-
				PTPRB:chr12:g.70609388C>T	-
				c.2886G>C	rs1282856643
**KMT2D*	NM_003482.4	c.511C>T	rs770105193	c.9614G>C	-
		c.12133C>T	-	c.9232G>C	-
				c.9186G>C	-
				c.8914G>A	-
EPPK1	NM_031308.4			c.6566C>A	-
				c.2843A>G	-

				c.13964G>A	rs1486151503
				c.3355G>A	rs200211342
				c.2558G>A	rs182198840
CCDC168	NM_001146197.3			c.12737C>A	-
				c.11379G>T	-
				c.11043G>C	-
				c.1718G>A	rs1298360300
				c.13597C>T	rs765904390
XIRP2	NM_152381.6	c.8730C>A	-	c.5210C>T	-
				c.1281G>C	-
				c.6911C>A	rs759805308
TMEM132C	NM_001136103.3	c.950C>T	-	TMEM132C:chr12:g.128543908G>A	-
		c.2665C>T	-		
		c.218G>A	rs545431831		
ZAN	NM_173059.3	c.236G>A	rs759815650	c.5081C>G	-
				c.5405G>C	-
THSD7A	NM_015204.3	c.890G>A	rs750574911	c.3605G>A	-
				c.389A>G	rs182889564
TFAP2A	NM_001032280.3			c.50_60del	-
				c.861dupA	-
				c.158C>T	-
*NBEA		c.6226C>T	rs937383651	c.6060G>A	-
	NM_015678.5			c.1572T>G	-
				**c.46C>	-
HCN1	NM_021072.4	c.145G>A	rs1214766208	c.134G>T	-
				c.99_147del	-
HUWE1	NM_031407.7	c.12610_12612del	-	c.12448G>A	-
		c.9274G>A	-		
*GTF2I	NM_031407.7			c.178G>C	-
				c.20C>G	-
				c.233A>G	-
CSMD2	NM_052896.5			c.6599G>A	-
				c.1241G>A	-
				c.3986G>C	-
				**c.3653A>C	-
				**c.8609C>A	rs983521648
DMXL2	NM_052896.5			c.1228C>G	-
				c.8972G>A	rs1457497323
				c.2980A>T	rs1402444713
APOB	NM_000384.3	c.4928C>T	rs1008238083	c.10813A>G	-
				c.11711A>T	rs1216474211
				**c.8359G>T	-
CACNA1C	NM_000719.7	c.1049C>T	-	c.4184G>T	-
				c.2579G>A	rs730880056
CAPN9	NM_006615.3	c.574G>A	rs536743386	CAPN9:chr1:g.230767487CA>TG	rs386640213
		c.430G>A	rs373142698	c.985G>A	rs373024313
TERT	NM_198253.3			c.2713G>A	-
				TERT:upstream	rs1561215364
				TERT:upstream	rs1242535815
LRP1	NM_002332.3	c.4945G>A	rs756702192	c.7237-6C>G	-
		c.9313G>A	rs1208542470	c.-60del-	-
				c.10595-1G>C	-
				LRP1:chr12:g.57179305C>T	-
MUC19	NM_173600.2			c.3521-3C>T	-
				MUC19:chr12:g.40517996C>A	-

				MUC19:chr12:g.40526387G>A	-
				c.23026+3G>A	rs571099944
				MUC19:chr12:g.40518774A>G	rs1192112733
ABI3BP	NM_001377332.1			c.2525-3C>G	-
				ABI3BP:upstream	-
				ABI3BP:chr3:g.100862455GGTTGCT>-	-
				c.1625G>A	rs202241381
				ABI3BP:chr3:g.100811849A>C	rs140918578
MYO15A	NM_016239.4			c.*32A>G	-
				MYO15A:chr17:g.18158466G>C	-
				c.1687C>T	rs763019528
				c.2447G>A	rs1219944687
CTNND2	NM_001332.4	c.1231C>T	rs1490647469	c.1246C>T	-
				CTNND2:chr5:g.11903747C>T	-
				CTNND2:chr5:g.11364627G>C	rs191668396
CASKIN1	NM_020764.4			c.2011G>A	-
				c.3355G>T	-
				CASKIN1:chr16:g.2178656GC>AA:p.?	-
				CASKIN1:chr16:g.2190209C>T	rs528686030
LRP1B	NM_018557.3	c.9121G>A	-	c.11258-1G>C	-
				c.12629A>G	rs1284354287
MTOR	NM_004958.4			c.5246G>A	-
				c.5156A>C	-
				MTOR:chr1:g.11212934G>A	rs181405432
**CIC*	NM_015125.5	c.1264C>T	-	CIC:chr19:g.42287248CC>TT:p.?	-
				CIC:chr19:g.42274618G>A	rs1237895323
				**c.2699-20T>G	-
				**c.419G>A	rs529183042
**FAM135B*	NM_015912.4	**c.3540+3A>G		FAM135B:chr8:g.138132825A>C	-
				FAM135B:chr8:g.138251097C>T	-
				c.2974T>A	rs374106478

*: cancer-associated genes found exclusively in Latin Americans; **: variants found exclusively in tumors unpaired with adjacent normal penile tissue.

Table S6. Main altered genes described in three previous studies in which whole-exome sequencing was performed in Penile Squamous Cell Carcinomas from Europeans [14], Asians [15], and North Americans [16], compared to Latin Americans (this study).

**European (n=27)**			**Asian (n=30)**			**North American (n=34)**			**Latin American (n=30)**	
**Genes**	**HPV *status***		**Genes**	**HPV *status***		**Genes**	**HPV *status***		**Genes**	**HPV *status***
	**Positive**	**Negative**		**Positive**	**Negative**		**Positive**	**Negative**		**Positive**
NBPF1	7	6	*FAT1*	1	3	*NOTCH1*	5	10	*NOTCH1*	10
FLG	1	3	*CASP8*	0	4	*FAT1*	2	10	*TERT*	9
XIRP2	1	2	*NOTCH1*	0	4	*TP53*	1	11	*FAT1*	9
SACS	1	1	*HRAS*	0	4	*TTN*	2	9	*TTN*	9
NUP210L	1	1	*PIK3CA*	1	2	*CDKN2A*	2	8	*TP53*	8
NSD1	0	3	*TP53*	1	2	*CASP8*	1	7	*CDKN2A*	7
MUC17	1	2	*FLG*	0	3	*MUC4*	3	4	*LRP1*	6
MGA	0	2	*SLITRK2*	1	2	*PIK3CA*	3	4	*RYR2*	6
KIT	1	1	*CCDC168*	0	3	*DNAH6*	2	4	*CASP8*	5
IL7R	2	1	*TRRAP*	1	2	*AHNAK2*	1	4	*FBXW7*	5
HRAS	3	1	*TTN*	0	3	*EP300*	3	2	*ITGA8*	5
HLA-B	1	4				*FBXW7*	0	5	*ABI3BP*	5
DNAH12	1	2				*LAMA1*	2	2	*HMCN2*	5
ZRANB3	0	1				*MUC17*	2	3	*MUC19*	5
ZNF180	0	1				*MUC2*	3	2	*AJUBA*	4

ZFHX3	1	1	*AK302511*	1	3	*CASKIN1*	4
WASF3	1	2	*ARP21*	4	0	*CTNND2*	4
TXNDC8	0	2	*BIRC6*	1	3	*TRPM2*	4
TSC1	1	2	*CACNA1C*	1	3	*SMARCA4*	4
TNFRSF14	1	1	*CSPG4*	3	1	*PTPRB*	4
TIMM17A	0	0	*FAT4*	1	3	*CR1*	4
TGFBR2	1	1	*FHAD1*	2	2	*PCLO*	4
TET2	1	1	*FRG1*	2	2	*MYO15A*	4
TDRD10	1	1	*FRY*	3	1	*USH2A*	4
STK19	1	0	*FSIP2*	2	2	*KMT2C*	4
SPEN	0	1	*GRIN2B*	2	2	*KRT6A*	3
SNX25	0	2	*KMT2B*	0	4	*TFAP2A*	3
SETDB1	2	1	*MYO18B*	2	2	*TMEM132C*	3
SELP	1	1	*PDE4DIP*	2	2	*HCN1*	3
PRDM1	0	2	*PKD1*	1	3	*RTN1*	3
OTUD7A	1	1				*CAPN9*	3
OR52N1	0	1				*PRDM9*	3
OR4A16	0	1				*PPP2R3A*	3
NTN4	1	1				*ARHGEF15*	3
NOTCH1	1	2				*PADI1*	3
NF1	1	2				*SEL1L2*	3
NCOR1	0	2				*GFPT2*	3
MYOCD	0	1				*ZNF831*	3
MORC4	0	1				*VPS41*	3
MLL3	3	6				*SEMA6D*	3
MICALCL	1	0				*GUCY2D*	3
ITPKB	1	0				*ARHGAP31*	3
HLA-A	1	1				*LLGL1*	3
FBXW7	2	1				*FAM135B*	3
FAM1666A	1	0				*AFF2*	3
EP300	0	1				*ATP10A*	3
DIS3	1	0				*CNTNAP5*	3
CTCF	1	0				*EPPK1*	3
CREBBP	1	1				*COL6A1*	3
COL5A3	1	2				*CEP162*	3
CHD4	1	2				*GTF2I*	3
CDKN2A	1	0				*ADAMTS12*	3
CASP8	2	1				*MCF2L*	3
C3orf70	0	1				*RRP12*	3
BRE	2	0				*CIC*	3
BCLAF1	1	0				*THSD7A*	3
ATM	1	0				*XIRP2*	3
ANK3	2	1				*KMT2D*	3
ALPK2	0	1				*MROH2A*	3
ALK	1	2				*CCDC168*	3
						PLCE1	3
						CHD5	3
						ZAN	3
						CACNA1C	3
						DMXL2	3
						COL11A1	3
						MTOR	3
						APOB	3
						ZZEF1	3
						NBEA	3
						RYR3	3
						ANK2	3
						CSMD2	3

PKHD1	3
MUC5AC	3
FRAS1	3
HUWE1	3
DNAH11	3
LRP1B	3
SSPOP	3
ADGRV1	3
OBSCN	3
SYNE1	3
NEB	3

Table S7: Genes with variants in untranslated regions (UTRs), and the miRNAs that binding by seed region.

Gene	miRNA	miRabel score	Variant alteration	Hybridization codon	LogitProb[1]	ΔGhybrid[2]	ΔGtotal[3]	Site access[4]
CARD11	*miR-93-5p*	0.0019	c.*18G>A	16-21	0.542	-16.900	-10.492	0.458
	miR-106b-5p	0.0042		16-21	0.501	-12.400	-5.992	0.458
	miR-20b-5p	0.0048		14-19	0.523	-19.000	-6.226	0.451
	miR-20b-5p	0.0048		14-18	0.504	-16.700	-3.871	0.346
CSMD3	*miR-1237-3p*	0.0059	c.-19T>C	16-21	0.557	-26.500	-7.866	0.231
	miR-1237-3p	0.0059		16-20	0.553	-18.600	-2.889	0.234
	miR-1237-3p	0.0059		16-21	0.543	-22.900	-2.955	0.243
	miR-224-5p	0.0372		19-25	0.511	-17.900	-0.541	0.297
	miR-224-5p	0.0372		19-24	0.501	-17.800	-0.608	0.327
	miR-132-3p	0.0434		15-19	0.521	-21.200	-3.702	0.249
KDR	*miR-331-3p*	0.0436	c.*4G>A	3-8	0.556	-22.900	5.722	0.095
				3-7	0.547	-20.200	2.942	0.137
TLX3	*miR-346*	0.0187	c.-35C>T	30-39	0.501	-20.300	-5.227	0.466

[1] probability of the site being a miRNA binding site as predicted by nonlinear logistic model; [2] measures of stability for miRNA-target hybrid as computed by *RNAhybrid*; [3] measures of the total energy change of the hybridization; [4] measures of structural accessibility as computed by the average probability of a nucleotide being single-stranded (i.e., unpaired) for the nucleotides in the predicted binding site.

Table S8. The top twenty KEGG pathways identified from a spectrum of major genes with somatic variants in HPV-associated Penile Squamous Cell Carcinomas from Latin Americans, ranked by FDR value.

ID	KEGG Pathway	Observed gene count	Background gene count	Strength	*FDR*	Genes
hsa05225	Hepatocellular carcinoma	9	160	1.56	1.18e-09	*PIK3CA, RB1, TP53, EGFR, TERT, PTEN, AKT2, CDKN2A* and *MYC*
hsa05165	Human papillomavirus infection	10	325	1.3	5.66e-09	*PIK3CA, RB1, TP53, EGFR, NOTCH1, TERT, CASP8, PTEN, ITGA8* and *AKT2*
hsa05214	Glioma	7	72	1.8	5.66e-09	*PIK3CA, RB1, TP53, EGFR, PTEN, AKT2* and *CDKN2A*
hsa05218	Melanoma	7	72	1.8	5.66e-09	*PIK3CA, RB1, TP53, EGFR, PTEN, AKT2* and *CDKN2A*
hsa05224	Breast cancer	8	145	1.56	5.66e-09	*PIK3CA, RB1, TP53, EGFR, NOTCH1, PTEN, AKT2* and *MYC*
hsa05206	MicroRNAs in cancer	8	160	1.51	9.31e-09	*PIK3CA, ERBB3, TP53, EGFR, NOTCH1, PTEN, CDKN2A, MYC*
hsa01522	Endocrine resistance	7	95	1.68	1.01e-08	*PIK3CA, RB1, TP53, EGFR, NOTCH1, AKT2* and *CDKN2A*

hsa05200	Pathways in cancer	11	517	1.14	1.01e-08	*PIK3CA*, *RB1*, *TP53*, *EGFR*, *NOTCH1*, *TERT*, *CASP8*, *PTEN*, *AKT2*, *CDKN2A* and *MYC*
hsa05213	Endometrial cancer	6	57	1.84	2.44e-08	*PIK3CA*, *TP53*, *EGFR*, *PTEN*, *AKT2* and *MYC*
hsa05166	Human T-cell leukemia virus 1 infection	8	211	1.39	4.61e-08	*PIK3CA*, *RB1*, *TP53*, *TERT*, *PTEN*, *AKT2*, *CDKN2A* and *MYC*
hsa05163	Human cytomegalovirus infection	8	218	1.38	5.37e-08	*PIK3CA*, *RB1*, *TP53*, *EGFR*, *CASP8*, *AKT2*, *CDKN2A* and *MYC*
hsa05223	Non-small cell lung cancer	6	68	1.76	5.37e-08	*PIK3CA*, *RB1*, *TP53*, *EGFR*, *AKT2* and *CDKN2A*
hsa05230	Central carbon metabolism in cancer	6	69	1.75	5.37e-08	*PIK3CA*, *TP53*, *EGFR*, *PTEN*, *AKT2* and *MYC*
hsa05212	Pancreatic cancer	6	73	1.73	6.31e-08	*PIK3CA*, *RB1*, *TP53*, *EGFR*, *AKT2* and *CDKN2A*
hsa04151	PI3K-Akt signaling pathway	9	350	1.22	6.49e-08	*PIK3CA*, *ERBB3*, *TP53*, *EGFR*, *ERBB4*, *PTEN*, *ITGA8*, *AKT2* and *MYC*
hsa05220	Chronic myeloid leukemia	6	75	1.72	6.49e-08	*PIK3CA*, *RB1*, *TP53*, *AKT2*, *CDKN2A* and *MYC*
hsa05226	Gastric cancer	7	144	1.5	6.57e-08	*PIK3CA*, *RB1*, *TP53*, *EGFR*, *TERT*, *AKT2* and *MYC*
hsa04218	Cellular senescence	7	150	1.48	8.15e-08	*PIK3CA*, *RB1*, *TP53*, *PTEN*, *AKT2*, *CDKN2A* and *MYC*
hsa04012	ErbB signaling pathway	6	83	1.67	9.61e-08	*PIK3CA*, *ERBB3*, *EGFR*, *ERBB4*, *AKT2* and *MYC*
hsa05160	Hepatitis C	7	156	1.47	9.61e-08	*PIK3CA*, *RB1*, *TP53*, *EGFR*, *CASP8*, *AKT2* and *MYC*
